# ST6Gal1 targets the ectodomain of ErbB2 in a site-specific manner and regulates gastric cancer cell sensitivity to trastuzumab

**DOI:** 10.1038/s41388-021-01801-w

**Published:** 2021-05-04

**Authors:** Henrique O. Duarte, Joana G. Rodrigues, Catarina Gomes, Paul J. Hensbergen, Agnes L. Hipgrave Ederveen, Arnoud H. de Ru, Stefan Mereiter, António Polónia, Elisabete Fernandes, José A. Ferreira, Peter A. van Veelen, Lúcio L. Santos, Manfred Wuhrer, Joana Gomes, Celso A. Reis

**Affiliations:** 1 i3S—Instituto de Investigação e Inovação em Saúde, Universidade do Porto, Porto, Portugal; 2 IPATIMUP—Institute of Molecular Pathology and Immunology of the University of Porto, Porto, Portugal; 3 ICBAS—Institute of Biomedical Sciences Abel Salazar, University of Porto, Porto, Portugal; 4 Leiden University Medical Center, Center for Proteomics and Metabolomics, Leiden, The Netherlands; 5 IPATIMUP Diagnostics, Department of Pathology, IPATIMUP, University of Porto, Porto, Portugal; 6 Experimental Pathology and Therapeutics Group, IPO-Porto Research Center, Portuguese Institute of Oncology, Porto, Portugal; 7 Department of Surgical Oncology, Portuguese Institute of Oncology, Porto, Portugal; 8 Faculty of Medicine, University of Porto, Porto, Portugal; 9 Present Address: IMBA, Institute of Molecular Biotechnology, Austrian Academy of Sciences, Vienna, Austria

**Keywords:** Proteomics, Gastric cancer, Glycobiology, Glycosylation, Prognostic markers

## Abstract

The clinical performance of the therapeutic monoclonal antibody trastuzumab in the treatment of ErbB2-positive unresectable gastric cancer (GC) is severely hampered by the emergence of molecular resistance. Trastuzumab’s target epitope is localized within the extracellular domain of the oncogenic cell surface receptor tyrosine kinase (RTK) ErbB2, which is known to undergo extensive *N*-linked glycosylation. However, the site-specific glycan repertoire of ErbB2, as well as the detailed molecular mechanisms through which specific aberrant glycan signatures functionally impact the malignant features of ErbB2-addicted GC cells, including the acquisition of trastuzumab resistance, remain elusive. Here, we demonstrate that ErbB2 is modified with both α2,6- and α2,3-sialylated glycan structures in GC clinical specimens. In-depth mass spectrometry-based glycomic and glycoproteomic analysis of ErbB2’s ectodomain disclosed a site-specific glycosylation profile in GC cells, in which the ST6Gal1 sialyltransferase specifically targets ErbB2 *N*-glycosylation sites occurring within the receptor’s trastuzumab-binding domain. Abrogation of ST6Gal1 expression reshaped the cellular and ErbB2-specific glycomes, expanded the cellular half-life of the ErbB2 receptor, and sensitized ErbB2-dependent GC cells to trastuzumab-induced cytotoxicity through the stabilization of ErbB dimers at the cell membrane, and the decreased activation of both ErbB2 and EGFR RTKs. Overall, our data demonstrates that ST6Gal1-mediated aberrant α2,6-sialylation actively tunes the resistance of ErbB2-driven GC cells to trastuzumab.

## Introduction

Gastric cancer (GC) remains a clinically challenging worldwide health burden, with over one million newly diagnosed cases every year [1, 2]. Furthermore, currently approved conventional chemotherapeutic modalities remain ineffective in improving the dismal prognosis of GC patients. The humanized monoclonal antibody (mAb) trastuzumab (Herceptin^®^) became the first personalized therapeutic agent approved by the Food and Drug Administration for the treatment of unresectable Human Epidermal Growth Factor Receptor 2 (ErbB2)-positive GC (10–15%) [2, 3]. By binding to the juxtamembrane portion of the subdomain IV of ErbB2’s extracellular region, trastuzumab acts primarily by preventing ErbB2 dimerization and promoting receptor internalization and intracellular degradation, which ultimately triggers cell cycle arrest and suppression of tumor cell proliferation. Unfortunately, the emergence of both intrinsic and acquired chemoresistance severely compromises the clinical performance of trastuzumab-based regimens.

The extracellular region of ErbB2 is composed of four structurally and functionally distinct subdomains, which tightly regulate receptor homo- or heterodimerization, and subsequent intracellular activation, and constitute well-known targets of extensive *N*-linked glycosylation [4, 5]. Moreover, glycans act as crucial regulators of key aspects of receptor tyrosine kinase (RTK) biology, such as correct folding and trafficking, membrane stability and residence time, dimerization capacity and signaling potential [6–9]. As for ErbB2, the receptor’s ectodomain harbors seven putative *N*-glycosylation residues (www.cbs.dtu.dk/services/NetNGlyc/). However, the detailed glycan composition of each of the receptor’s glycosylation site (glycosite), as well as the functional implications that distinct glycosylation signatures may bear on the regulation of ErbB2 biology within gastric tumors, including the sensitivity to trastuzumab-based therapy, require further elucidation.

We have previously identified α2,6-linked sialic acid (α2,6NeuAc), a cancer-associated negatively charged monosaccharide residue, as a major glycan signature within the receptor’s glycosylation landscape in ErbB2-driven GC cells [8]. The terminal addition of α2,6NeuAc to the antennae of *N*-linked glycan chains is accomplished by the β-galactoside α2,6-sialyltranferase 1 (ST6Gal1) Golgi-residing glycosyltransferase. ST6Gal1 marked overexpression in multiple human cancers (including gastric), and the concomitant upregulation of α2,6-sialylation in specific target proteins, have been extensively associated with increased tumor invasive and metastatic capacities, as well as poor patient clinical outcome [10–14].

Here, we validate ErbB2 as an in vivo molecular carrier of both α2,3- and α2,6-sialylated *N*-glycan structures in gastric tumor clinical specimens. In addition, silencing of the *ST6GAL1* gene prompted the enrichment in both terminal α2,3-linked sialic acid (α2,3NeuAc)- and fucose-containing glycan species at the cell surface of ErbB2-dependent GC cells, and triggered a glycosite-specific reshaping of the ErbB2 glycome. This was characterized by the marked enrichment in multi-fucosylated species of both N530 and N629 glycosites, both occurring within ErbB2’s trastuzumab-binding domain. Importantly, *ST6GAL1* knockout (K.O.) ErbB2-addicted GC cells displayed a reduced ErbB2 degradation rate, and enhanced sensitivity to trastuzumab-induced cytotoxicity, supported by a decreased ability to sustain ErbB2 and epidermal growth factor receptor (EGFR) intracytoplasmic phosphorylation, and increased stabilization of ErbB dimeric compositions at the cell membrane.

## Results

### ErbB2 is a molecular carrier of α2,6- and α2,3-sialylated glycan species in gastric carcinomas

**Fig. 1:**
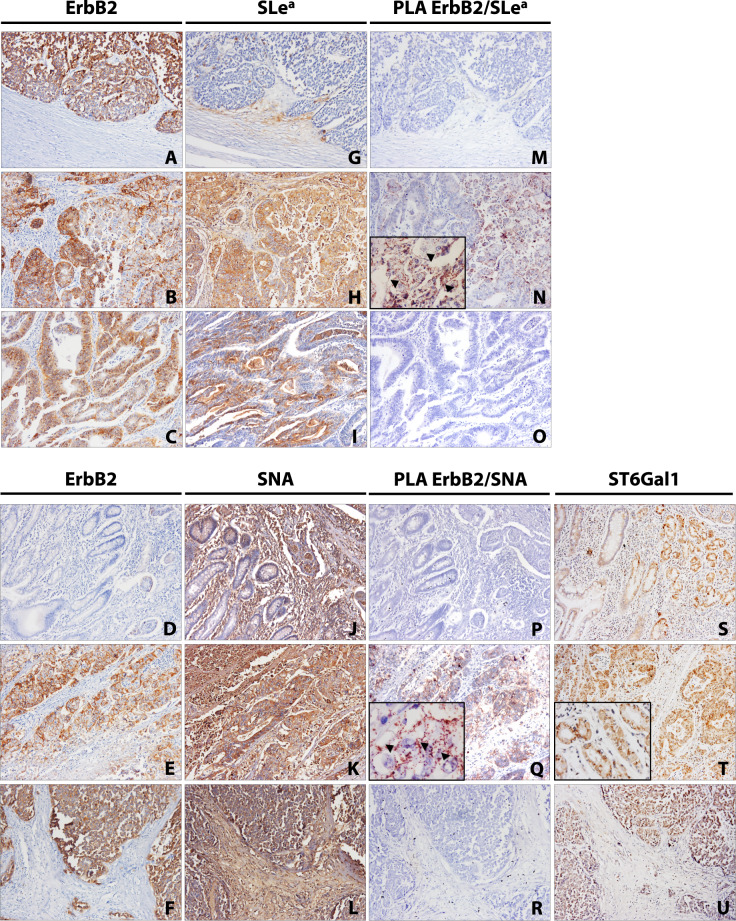
Expression of ErbB2 sialylated glycoforms in intestinal-type gastric carcinoma. Representative individual (immuno)histochemical detection of: ErbB2 (**A**–**F**), sialyl Lewis a (SLe^a^) (**G**–**I**), and *Sambucus nigra* agglutinin (SNA) (α2,6-linked sialic acid (α2,6NeuAc)) (**J**–**L**) in gastric carcinoma tissue sections; Parallel in situ brightfield Proximity Ligation Assay (PLA) analysis of ErbB2-SLe^a^ (**M**–**O**) and ErbB2-SNA (**P**–**R**) molecular proximity; representative individual immunohistochemical detection of ST6Gal1 (**S**–**U**) in gastric carcinoma tissue sections; images were acquired under ×200 and ×630 (inserts) magnifications.

Like most membrane-anchored proteins, ErbB2 functionality is tightly regulated by the receptor’s *N*-linked glycan repertoire [8, 15]. We have previously identified ErbB2 as an in vitro carrier of both α2,3- (sialyl Lewis a (SLe^a^)) and α2,6NeuAc glycan epitopes in *ERBB2*-amplified GC cells [8]. Still, the expression of specific ErbB2 sialylated glycoforms in GC tissues has yet to be fully elucidated. To evaluate the expression of both ErbB2 sialylated glycoforms in GC, the molecular proximity between ErbB2 and SLe^a^ and α2,6NeuAc was detected through in situ brightfield Proximity Ligation Assay (PLA) in gastric tumor tissues, using the CA 19-9 mAb and the *Sambucus nigra* agglutinin (SNA), respectively. ErbB2 expression was evaluated by immunohistochemistry in a retrospective series of 173 stage I–IV gastric carcinomas (Fig. 1A–F). Of those, 19 (11%) intestinal-type gastric adenocarcinomas were found positive for ErbB2 expression. The individual expression of SLe^a^ (Fig. 1G–I) and α2,6NeuAc (SNA) (Fig. 1J–L) was assessed by (immuno)histochemistry in ErbB2-positive cases, and subsequent PLA characterization was performed. Table S1 summarizes the clinicopathological information of the selected ErbB2-positive GC patients. A positive punctuated and membrane-confined PLA signal, restricted to tissue regions where single biomarker co-localization was observed, was detected in 58% of the evaluated GC cases for both SLe^a^ (Fig. 1M–O) and α2,6NeuAc (SNA) (Fig. 1P–R), portraying, for the first time, ErbB2 as an in vivo carrier of sialylated glycan structures. In the case of α2,6NeuAc, ST6Gal1 immunohistochemical expression was robustly co-localized with positive PLA signals (Fig. 1S–U).

### ST6Gal1 mediates the cell surface α2,6-sialylation of ErbB2-driven gastric cancer cells

**Fig. 2:**
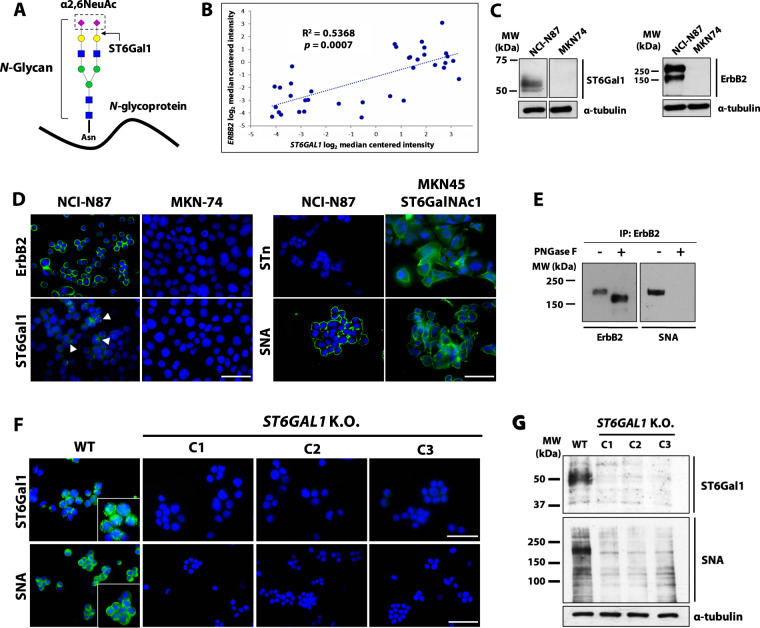
*ST6GAL1* K.O. abrogates the cell surface expression of α2,6-linked sialic acid in ErbB2-positive gastric cancer cells. **A** Schematic representation of terminal α2,6-sialylation (α2,6NeuAc) of *N*-glycosylated proteins via ST6Gal1; **B** Positive Spearman’s rank correlation between the *ERBB2* and *ST6GAL1* transcript expression in gastric cancer (GC) patients from the DErrico dataset available at the Oncomine^TM^ database; **C** Western blot analysis of ErbB2 and ST6Gal1 expression in intestinal-type GC cell lines; **D** Immunofluorescence detection of ErbB2, ST6Gal1, and total (*Sambucus nigra* agglutinin (SNA)) and *O*-linked (sialyl Tn (STn)) α2,6-linked sialic acid (α2,6NeuAc) in intestinal-type GC cell lines; DAPI nuclear staining is shown in blue; The scale bar corresponds to 60 μm; **E** Western blot analysis of ErbB2 and α2,6NeuAc following receptor immunoprecipitation from NCI-N87 whole cell lysates and Peptide *N*-Glycosidase F (PNGase F) digestion; **F** Immunofluorescence detection of ST6Gal1 and its glycan product (α2,6NeuAc) in NCI-N87 WT and *ST6GAL1* K.O. cells; DAPI nuclear staining is shown in blue; The scale bar corresponds to 60 and 40 μm, in the ST6Gal1 and SNA panels, respectively; **G** Western blot analysis of ST6Gal1 and α2,6NeuAc (SNA) expression in NCI-N87 WT and *ST6GAL1* K.O. cells; C1 clone 1, C2 clone 2, C3 clone 3.

Despite having identified ErbB2 as a protein carrier of α2,6NeuAc in the ErbB2-positive NCI-N87 GC cell line [8], this glycan epitope can occur in the context of either *O*-glycan or *N*-glycan chains (via ST6Gal1, Fig. 2A). Despite ErbB2 harboring multiple predicted *O*-glycosylation sites, none has yet been experimentally demonstrated. Bioinformatic analysis of a cohort of GC patients disclosed a positive correlation between the *ERBB2* and *ST6GAL1* genes (Fig. 2B). Furthermore, marked and absent ST6Gal1 expression were also observed in the intestinal-type ErbB2-positive NCI-N87 and ErbB2-negative MKN-74 GC cell lines, respectively (Fig. 2C, D—left panel). To further dismiss the presence of α2,6NeuAc-modified *O*-glycans, immunofluorescent detection of the *O*-linked GalNAc-α2,6NeuAc sialyl Tn (STn) antigen, and parallel detection of total α2,6NeuAc, by SNA reactivity, were performed in the ErbB2-positive NCI-N87 cells (Fig. 2D—right panel). The MKN45 GC cell line stably transfected with the *N*-acetylgalactosaminide α2,6-sialyltransferase 1 (ST6GalNAc1) sialyltransferase was used as a positive control of STn overexpression [16]. While both cell lines exhibited marked SNA staining, no membranous expression of STn was detected in NCI-N87 cells. Furthermore, digestion of immunoprecipitated ErbB2 with Peptide *N*-Glycosidase F (PNGase F), which cleaves all *N*-linked glycan chains from a protein backbone, followed by SNA lectin blot analysis, revealed the complete abrogation of the SNA signal and the concomitant shift on ErbB2 molecular weight (MW) (Fig. 2E). We therefore conclude that ErbB2 carries α2,6NeuAc epitopes within *N*-glycan chains in GC cells.

The overexpression and dysregulated activity of the ST6Gal1 leads to the aberrant terminal α2,6-sialylation of *N*-glycan chains, and has been consistently associated with human malignancy [12–14]. To assess the functional impact of *N*-linked α2,6-sialylation, the *ST6GAL1* gene was silenced in the ErbB2-driven NCI-N87 GC cell line, using the CRISPR-Cas9 system. The guide RNA (gRNA) insertion/deletion (indel) signature at the *ST6GAL1* locus of individual isogenic clones was determined using a previously established PCR-based indel detection by amplicon analysis method (Fig. S1A) [17, 18], and was further validated by direct Sanger sequencing (Fig. S1B) and with the tracking of indels by decomposition (TIDE) bioinformatic tool (Fig. S1C) [19]. Three independent clones (C1-3) were selected for further experiments. A concomitant significant downregulation of the *ST6GAL1* mRNA levels in all the three K.O. clones was observed (Fig. S1D). Immunofluorescence staining of ST6Gal1 was performed, revealing an intense Golgi-like punctuated staining confined to the cytoplasmic compartment of wild-type (WT) cells, whereas no signal was observed in *ST6GAL1* K.O. cells (Fig. 2F—upper panel). These observations were further corroborated by western blot analysis (Fig. 2G). NCI-N87 WT cells showed intense SNA reactivity at the cell surface, as opposed to *ST6GAL1 K.O*. clones, for which no signal was detected (Fig. 2F—lower panel). SNA lectin blot and flow cytometry analysis, for the detection of α2,6-sialylated species, exhibited a marked decrease in SNA reactivity in *ST6GAL1* K.O. clones (Figs. 2G, S1E, respectively). Silencing of *ST6GAL1* did not produce significant effects on cell proliferation (Fig. S1F).

### *ST6GAL1* K.O. reshapes the terminal antenna decoration pattern of *N*-glycan chains

**Fig. 3:**
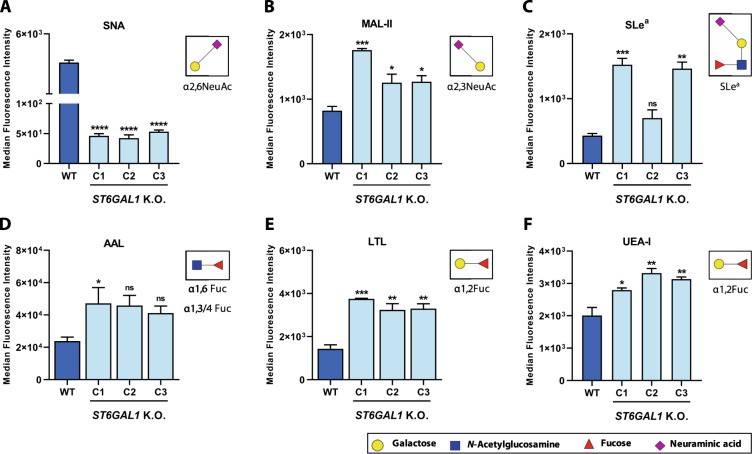
*ST6GAL1* K.O. upregulates the cell surface expression of alternative glycan capping motifs. Cell surface flow cytometric staining of NCI-N87 WT and *ST6GAL1* K.O. ErbB2-positive cells with the (**A**) *Sambucus nigra* agglutinin (SNA) recognizing α2,6-linked sialic acid (α2,6NeuAc); **B** *Maackia amurensis* lectin II (MAL-II) recognizing α2,3-linked sialic acid (α2,3NeuAc); **C** CA 19.9 monoclonal antibody (mAb) recognizing sialyl Lewis a (SLe^a^); **D** *Aleuria aurantia* lectin (AAL) recognizing α1,6- and α1,3/4-linked fucose (α1,6Fuc and α1,3/4Fuc); **E** *Lotus tetragonolobus* lectin (LTL) recognizing α1,2-linked fucose (α1,2Fuc); **F** *Ulex europaeus* agglutinin I (UEA-I) recognizing α1,2-linked fucose (α1,2Fuc); Comparisons were made using one-way ANOVA analysis of variance (*n* = 3; mean ± SD; *p < 0.05; **p < 0.01; ***p < 0.001); n.s. nonsignificant, C1 clone 1, C2 clone 2, C3 clone 3.

The nonreducing end of *N*-glycan chains can be modified with sialic acid, through either α2,6- or α2,3-linkages, and neutral fucosylated motifs [20]. Thus, α2,3-sialylation and fucosylation could represent alternative mechanisms for the terminal capping of *N*-glycan antennae, in the absence of ST6Gal1 activity. Since *ST6GAL1* K.O. ErbB2-positive GC cells lack the membranous expression of α2,6NeuAc (Fig. 3A), the cell surface levels of terminal α2,3NeuAc and fucosylated antigens were assessed, by flow cytometry, in WT and *ST6GAL1* K.O. cells. Two α2,3NeuAc-recognizing molecules were used: the *Maackia amurensis* lectin II (MAL-II), which harbors affinity towards α2,3NeuAc-containing glycan epitopes; and the CA-19.9 mAb, which recognizes the cancer-associated SLe^a^ antigen, previously shown to be carried by ErbB2 [8]. In all three K.O. clones, a significant increase in the MAL-II staining was detected, indicating the upregulation of α2,3NeuAc cell surface expression (Fig. 3B). The same trend was observed in regard to SLe^a^ expression (Fig. 3C). Additionally, three lectins showing distinct specificities towards fucose residues were applied: the *Aleuria aurantia* lectin (AAL), which binds to overall α-linked fucose-containing glycan chains, and the *Lotus tetragonolobus* lectin (LTL) and *Ulex europaeus* agglutinin I (UEA-I), which bind with high affinity to α1,2-linked antenna fucose (α1,2Fuc) moieties. Although AAL staining reached a significant increase solely in C1, the cell surface expression of both LTL and UEA-I ligands was significantly increased in all *ST6GAL1* K.O. clones, when compared to the WT cells, indicating an upregulation of terminally fucosylated *N*-glycan chains (Fig. 3D–F, respectively). Overall, the absence of α2,6NeuAc carbohydrate motifs drives the upregulation of alternative glycan epitopes that compete for the terminal capping of the antennae of *N*-glycan chains.

### ST6Gal1 targets ErbB2 trastuzumab-binding domain in a glycosite-specific manner

**Fig. 4:**
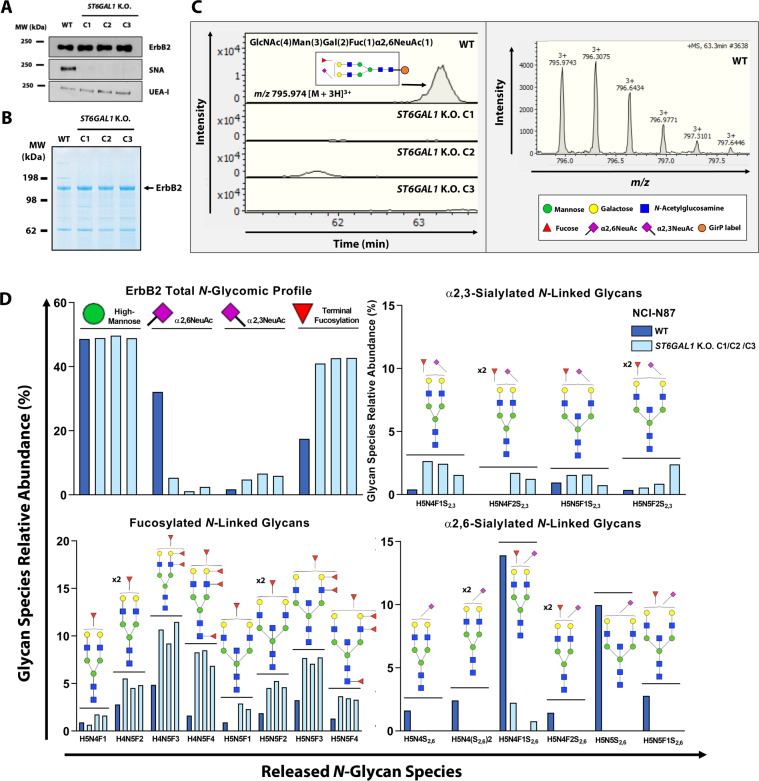
*ST6GAL1* K.O. triggers the competitive modification of ErbB2 *N*-glycan antennae with multi-fucosylated species. **A** *Sambucus nigra* agglutinin (SNA) and *Ulex europaeus* agglutinin I (UEA-I) lectin blot analysis of ErbB2 carrying α2,6-linked sialic acid (α2,6NeuAc) and α1,2-linked fucose (α1,2Fuc), respectively, following receptor immunoprecipitation from NCI-N87 WT and *ST6GAL1* K.O. whole cell lysates; **B** Colloidal Blue gel staining of immunoprecipitated WT and *ST6GAL1* K.O. ErbB2. In all samples, the indicated band corresponding to ErbB2 was excised and further processed for glycomic analysis; **C** Illustrative example of peak quantification and isotopic distribution of an α2,6NeuAc-containing *N*-glycan species in the electropherograms of WT and *ST6GAL1* K.O. ErbB2. Following receptor immunoprecipitation, in-gel Peptide *N*-Glycosidase F (PNGase F) digestion of the excised bands was performed to achieve total *N*-glycan release, followed by sialic acid derivatization, *N*-glycan purification and labeling with the Girard’s reagent P (GirP), and analysis by capillary electrophoresis–electrospray ionization-mass spectrometry (CE–ESI-MS); **D** Relative quantification of the released *N*-glycan species from WT and *ST6GAL1* K.O. ErbB2; C1 clone 1, C2 clone 2, C3 clone 3.

We further investigated how the silencing of *ST6GAL1* would remodel the ErbB2-specific glycome. Immunoprecipitated ErbB2 was probed with the α2,6NeuAc- and α1,2Fuc-recognizing SNA and UEA-I lectins, respectively. Regarding SNA, a clear band matching the expected MW of the fully glycosylated receptor was observed in the WT sample, confirming ErbB2 as a carrier of α2,6NeuAc moieties (Fig. 4A). As for *ST6GAL1* K.O. ErbB2, no SNA reactivity was detected. In parallel, *ST6GAL1* K.O. ErbB2 exhibited an increase in UEA-I reactivity in comparison to WT ErbB2, indicating an upregulation of terminally fucosylated glycan species. To fully assess these structural alterations, mass spectrometry (MS)-based glycomic and glycoproteomic workflows were implemented (Fig. S2).

The glycomic analysis of ErbB2 was performed following the release of total *N*-glycan species from the receptor through the in-gel digestion of the excised bands with PNGase F (Fig. 4B). Figure 4C depicts an illustrative example of the obtained electropherograms corresponding to an α2,6NeuAc-containing mono-sialylated (mS) biantennary *N*-glycan structure (GlcNAc(4)Man(3)Gal(2)α2,6NeuAc(1)). This species was solely detected in WT ErbB2, but not in *ST6GAL1* K.O. ErbB2. The overall glycomic profile of both WT and *ST6GAL1* K.O. ErbB2 confirmed that the loss of ST6Gal1 function leads to no alterations in the relative amount of detected oligomannosidic species, which do not constitute substrates of ST6Gal1 (Fig. 4D—upper left panel). As for complex *N*-glycans, a drastic loss of all α2,6NeuAc-modified species was confirmed in *ST6GAL1* K.O. ErbB2 (Fig. 4D—lower right panel). Furthermore, *ST6GAL1* K.O. triggered the upregulation of terminally mono—(mF), di—(dF), tri—(tF), and tetra-fucosylated (ttF) *N*-glycan species (Fig. 4D—lower left panel). A slight increase in the relative amounts of α2,3NeuAc-containing structures was consistently observed across all *ST6GAL1* K.O. ErbB2 samples (Fig. 4D—upper right panel).

**Fig. 5:**
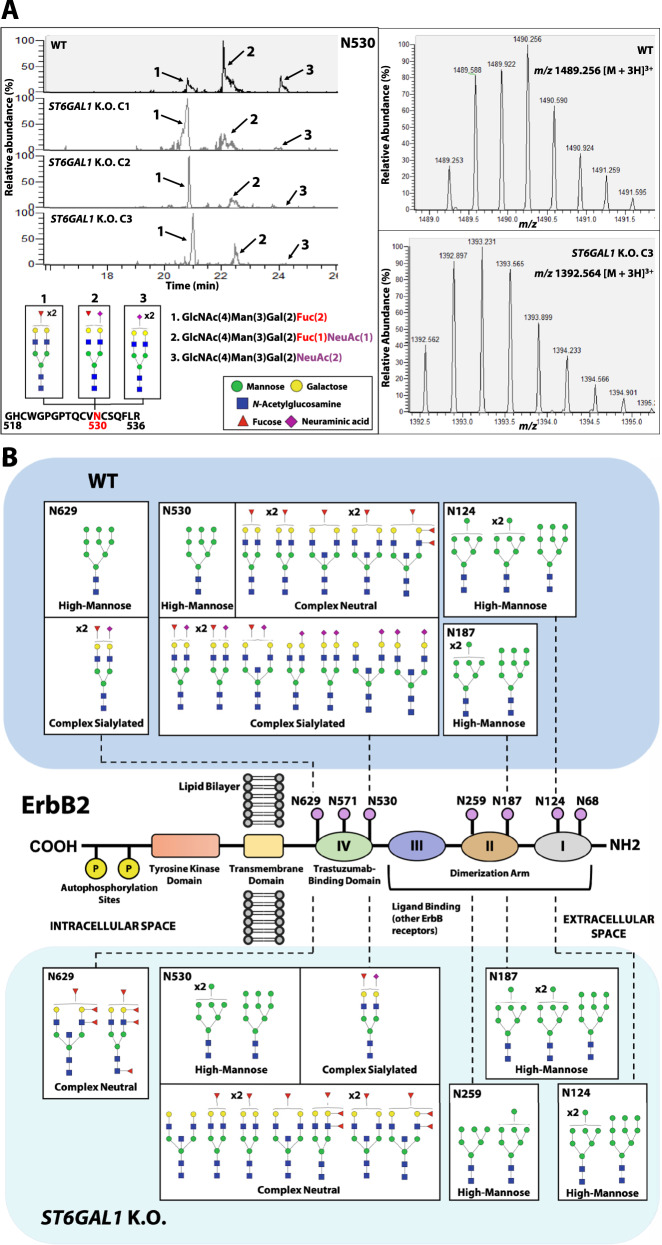
ST6Gal1 specifically targets glycosylation sites within ErbB2 trastuzumab-binding domain. **A** Left panel—combined extracted ion chromatogram of the N530 glycopeptide modified with: a di-fucosylated (dF) biantennary *N*-glycan (peak 1, peptide + GlcNAc(4)Man(3)Gal(2)Fuc(2), ion at *m/z* 1392.564, [M + 3H]^3+^), a mono-sialylated mono-fucosylated (mSmF) biantennary *N*-glycan (peak 2, peptide + GlcNAc(4)Man(3)Gal(2)Fuc(1)NeuAc(1), ion at *m/z* 1440.910, [M + 3H]^3+^) and di-sialylated (dS) biantennary *N*-glycan biantennary *N*-glycan (peak 3, peptide + GlcNAc(4)Man(3)Gal(2)NeuAc(2) ion at *m/z* 1489.256, [M + 3H]^3+^) in WT and *ST6GAL1* K.O. C3 ErbB2. Right panels—isotopic distribution of the N530 glycopeptide modified with a dS biantennary *N*-glycan in WT ErbB2, and with a dF biantennary *N*-glycan in *ST6GAL1* K.O. C3 ErbB2; **B** Schematic representation of glycosylation site assignment and structural characterization in WT and *ST6GAL1* K.O. ErbB2; C1 clone 1, C2 clone 2, C3 clone 3.

The peptidic sequence of ErbB2 contains seven putative *N*-glycosylation target sites: N68 and N124 in subdomain I, N187 and N259 in subdomain II, and N530, N571 and N629 located within subdomain IV, which harbors the trastuzumab-binding epitope [21]. To comprehensively map and characterize the glycan composition of specific ErbB2 glycosites, a MS-based glycoproteomics strategy was used (Fig. S2). ErbB2 was identified as the major glycosylated protein in all excised bands (Fig. 4B). Protein coverage ranged from 70 to 77% and from 60 to 66% in the first and second biological replicate, respectively. A semiquantitative approach based on glycopeptide peak intensity was performed to assess the full extent of glycoproteomic alterations, using specific glycopeptide *m/z* traces. Out of the seven predicted ErbB2 *N*-glycosylation sites, five were assigned and had their glycosylation status characterized (Table S2). The glycosylation status of both N68 and N571 glycosites was not assessed, due to insufficient protein sequence coverage of the respective glycopeptide regions. Residues N530 and N629 of the WT receptor, occurring within ErbB2 trastuzumab-binding domain, were found to be the only ones carrying terminally sialylated and fucosylated complex *N*-glycans, besides oligomannosidic species. Thus, the glycan repertoire of the aforementioned sites was found to most significantly differ between WT and *ST6GAL1* K.O. ErbB2. Figure 5A depicts an illustrative example of the site-specific glycosylation shift occurring at N530. In WT ErbB2, the glycopeptide 518-GHCWGPGPTQCVNCSQFLR-536 was found to be heterogeneously occupied by dF (GlcNAc(4)Man(3)Gal(2)Fuc(2), *m/z* 1392.564, [M + 3H]^3+^), mono-sialylated mono-fucosylated (mSmF) (GlcNAc(4)Man(3)Gal(2)Fuc(1)NeuAc(1), *m/z* 1440.910, [M + 3H]^3+^), and di-sialylated (dS) (GlcNAc(4)Man(3)Gal(2)NeuAc(2), *m/z* 1489.256, [M + 3H]^3+^) biantennary complex *N*-glycan species (peaks 1, 2 and 3 of Fig. 5A—upper panel, respectively; Fig. S3A). The glycopeptide carrying the dF neutral glycan chains was the first to elute, followed by the ones modified with one (mSmF) and two (dS) sialic acid moieties. In the WT ErbB2, the mSmF-modified glycopeptide exhibited the highest peak intensity, followed by the dF- and dS-modified glycopeptides. In all *ST6GAL1* K.O. ErbB2 samples, however, only a residual signal could be detected for the dS-modified glycopeptide (peak 3), indicating the absence of the dS glycan. Moreover, in *ST6GAL1* K.O. ErbB2, the same glycopeptide carrying dF glycans showed the highest peak intensity. Fucosylation can either occur on the innermost *N*-acetylglucosamine (GlcNAc) of the *N*-glycan core, or at the antennae linked to a terminal galactose (Gal) or GlcNAc residue. The collision-induced dissociation (CID) MS/MS spectrum of the *ST6GAL1* K.O. glycopeptide further supports the presence of terminal fucose residues, rather than at the *N*-glycan core (Fig. S3A—lower panel). In addition, the K.O. of *ST6GAL1* led to the emergence of highly abundant N530 glycopeptides carrying both tF or ttF biantennary complex *N*-glycan species, which could not be detected in the WT ErbB2 sample (Fig. S3B). A similar shift in glycosylation was observed in glycosite N629. Figure 5B schematically illustrates the mapped and characterized glycosylation sites within functionally distinct subdomains of the receptor’s extracellular region. Glycosites N124, N187, and N259 were found to carry *N*-glycan chains exclusively of the high-mannose subtype (Man6-Man9), and showed no differences between WT and *ST6GAL1* K.O. ErbB2, with the exception of N259, which was solely detected in *ST6GAL1* K.O. ErbB2.

Using the same MS-based approach on a fresh-frozen tissue section of an ErbB2-positive gastric adenocarcinoma, the N124 glycosylation site was successfully validated and found to be occupied by Man9 glycan species, in agreement with observations made for GC cell line-derived ErbB2 (Fig. S4, Table S2).

### *ST6GAL1* K.O. sensitizes ErbB2-driven gastric cancer cells to trastuzumab-induced cytotoxicity

**Fig. 6:**
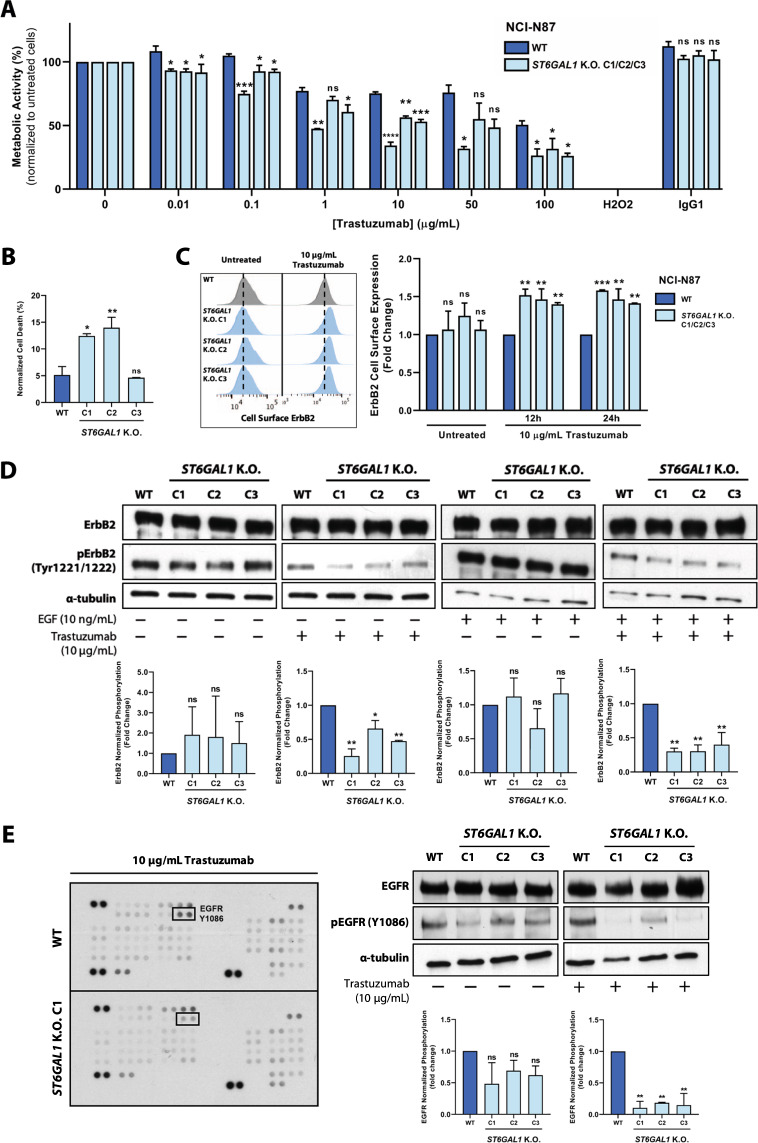
*ST6GAL1* K.O. sensitizes ErbB2-positive gastric cancer cells to trastuzumab-induced cytotoxicity. **A** Resazurin assay for the measurement of the metabolic activity of NCI-N87 WT and *ST6GAL1* K.O. ErbB2-positive gastric cancer (GC) cells following the treatment with increasing doses of trastuzumab; H_2_O_2_—positive control; IgG1—trastuzumab isotype control; **B** Assessment of trastuzumab-induced cell death through the annexin V/propidium iodide (PI) staining of WT and *ST6GAL1* K.O. cells treated with 10 μg/mL trastuzumab for 120 h; **C** Quantification of ErbB2 cell surface expression by trastuzumab-binding in WT and *ST6GAL1* K.O. cells treated with 10 μg/mL trastuzumab for 12 and 24 h; **D** Western blot analysis and α-tubulin-normalized quantification of ErbB2 intracytoplasmic phosphorylation in WT and *ST6GAL1* K.O. cells treated with 10 μg/mL trastuzumab for 120 h and stimulated with 10 ng/mL EGF; **E** Analysis of the phosphorylation status of 43 human kinases in NCI-N87 WT and *ST6GAL1* K.O. cells treated with 10 μg/mL trastuzumab for 120 h; Western blot analysis and α-tubulin-normalized quantification of EGFR intracytoplasmic phosphorylation for array target validation in NCI-N87 WT and *ST6GAL1* K.O. cells treated with 10 μg/mL trastuzumab for 120 h; Comparisons were made using one-way ANOVA analysis of variance (*n* = 3; mean ± SD; *p < 0.05; **p < 0.01; ***p < 0.001); n.s. nonsignificant, C1 clone 1, C2 clone 2, C3 clone 3.

Following the observation of the above-described ErbB2 glycomic alterations, the functional impact of *ST6GAL1* K.O. on the response of ErbB2-positive GC cells to trastuzumab-based therapy was assessed. When challenged with a wide range of mAb concentrations, *ST6GAL1* K.O. cells exhibited an increased sensitivity to trastuzumab, as shown by the significant downregulation of their metabolic activity (Fig. 6A). In parallel, a treatment with 10 µg/mL trastuzumab induced significantly higher cell death in two *ST6GAL1* K.O. clones, as showed by annexin V/propidium iodide (PI) staining (Fig. 6B). To understand the mechanistic basis underlying the observed phenotype, we analyzed the membrane-confined expression of ErbB2, through the cell surface staining of both WT and *ST6GAL1* K.O. cells with trastuzumab, following a 12 and 24 h treatment period with 10 µg/mL trastuzumab. Interestingly, at both time-points, treated *ST6GAL1* K.O. cells exhibited significantly higher levels of membrane-anchored ErbB2, indicating a prolonged stabilization of the ErbB2-trastuzumab complex at the cell surface (Fig. 6C). No differences in ErbB2 cell surface detection were observed in untreated cells. We further analyzed ErbB2 intracytoplasmic phosphorylation in NCI-N87 WT and *ST6GAL1* K.O. cells treated with 10 µg/mL trastuzumab and stimulated with 10 ng/mL EGF, an EGFR natural peptidic ligand. Following the treatment with trastuzumab, *ST6GAL1* K.O. clones exhibited a marked downregulation of ErbB2 intracellular activation, when compared with the WT cell line (Fig. 6D). No differences were observed in the absence of trastuzumab treatment, regardless of ligand stimulation. These results are consistent with the observed increase in sensitivity to trastuzumab-induced cytotoxicity and higher retention time of the ErbB2-trastuzumab complex at the plasma membrane, which precludes RTK intracellular activation and downstream mitogenic signaling. Finally, we used a human phospho-kinase array to uncover additional changes in the phosphorylation levels of 43 human kinases in whole cell lysates collected from WT and *ST6GAL1* K.O. C1, following the treatment with 10 µg/mL trastuzumab (Fig. S5A). EGFR, another cancer-associated member of the ErbB RTK receptor family, was found to be significantly less activated in *ST6GAL1* K.O. cells, upon treatment with trastuzumab (Fig. 6E). No differences in EGFR phosphorylation were observed in untreated cells (Fig. S5B, C). This result was further validated in the remaining two *ST6GAL*1 K.O. clones (Fig. 6E).

### *ST6GAL1* K.O. extends ErbB2 half-life and potentiates the trastuzumab-induced stabilization of ErbB dimers at the cell surface

**Fig. 7:**
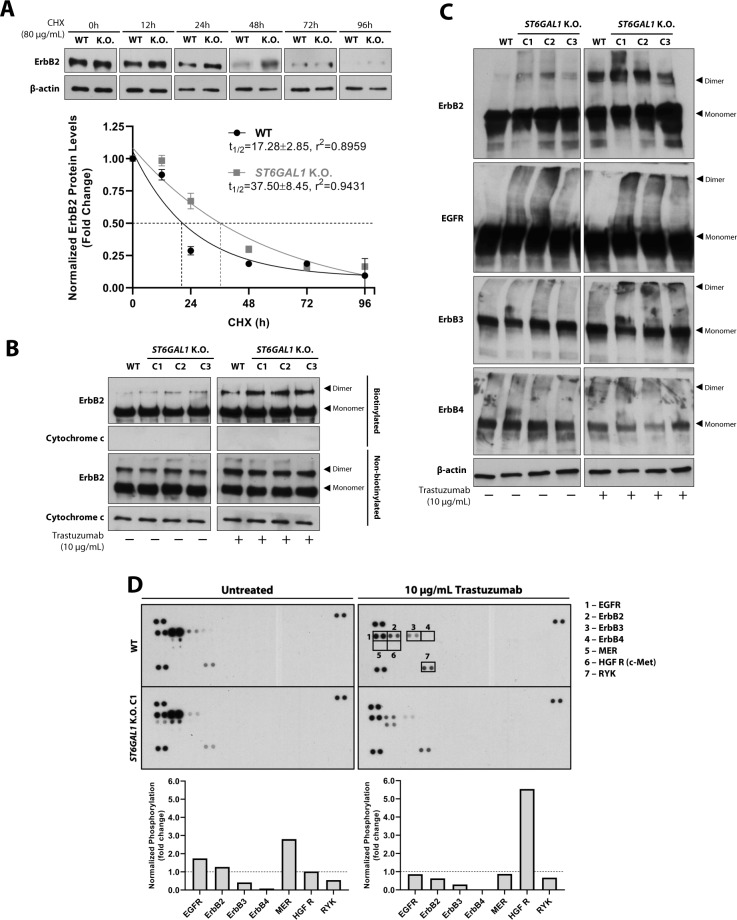
*ST6GAL1* K.O. increases ErbB2 protein half-life and potentiates the trastuzumab-induced stabilization of ErbB dimers at the cell membrane. **A** Western blot analysis of ErbB2 in whole cell lysates of NCI-N87 WT and *ST6GAL1* K.O. C1 cells after blocking protein synthesis with 80 μg/mL cycloheximide (CHX) for 0, 12, 24, 48, 72, and 96 h. ErbB2 protein half-life was calculated based on the density of the β-actin-normalized ErbB2 Western blot bands, using the one phase exponential decay function, and was defined as the time required for the ErbB2 protein to reach 50% of its initial level; *n* = 3 (mean ± SD); **B** Western blot analysis of biotinylated membrane-bound and non-biotinylated cytoplasmic ErbB2 from NCI-N87 WT and *ST6GAL1* K.O. cells treated with 10 μg/mL trastuzumab for 24 h. The mitochondrial marker cytochrome c was used as a control for non-biotinylated proteins from the cytoplasmic subcellular compartment; **C** Western blot analysis of ErbB2, EGFR, ErbB3, and ErbB4 dimerization in whole cell lysates of NCI-N87 WT and *ST6GAL1* K.O. cells cross-linked with bis(sulfosuccinimidyl)suberate (BS3), following treatment with 10 μg/mL trastuzumab for 24 h; **D** Analysis and quantification of the phosphorylation levels of 49 cancer-related cell membrane receptor tyrosine kinases (RTKs) in NCI-N87 WT and *ST6GAL1* K.O. cells treated with 10 μg/mL trastuzumab for 120 h.

Given that trastuzumab-treated NCI-N87 *ST6GAL1* K.O. cells exhibited increased cell surface staining of ErbB2, a cycloheximide (CHX) protein blocking assay was performed to evaluate possible differences in the protein half-life of WT and *ST6GAL1* K.O. ErbB2. Briefly, NCI-N87 WT and *ST6GAL1* K.O. cells were treated for 0–96 h with CHX protein synthesis inhibitor, and quantification of total ErbB2 protein levels was performed (Fig. 7A). The half-life of ErbB2 was considered the time-point at which CHX treatment reduced the receptor’s protein levels to half of those observed in untreated cells, and was estimated using the one phase exponential decay function. *ST6GAL1* K.O. cells depicted an extended ErbB2 half-life (37.50 ± 8.45), when compared to the WT (17.28 ± 2.85). To further dissect the increased ErbB2 cell surface detection in trastuzumab-treated *ST6GAL1* K.O. cells, we assessed the individual contributions of membrane-bound and cytoplasmic ErbB2 in NCI-N87 WT and *ST6GAL1* K.O. cells treated with 10 µg/mL trastuzumab by performing a streptavidin pull-down of biotinylated cell surface proteins. The detection of the mitochondrial marker cytochrome c was used as a control for non-biotinylated cytoplasmic proteins. Although no significant differences were observed for the 185 kDa ErbB2 monomer, *ST6GAL1 K.O*. cells exhibited an increased stabilization of the receptor’s dimeric form, which became even more apparent upon the treatment with trastuzumab (Fig. 7B). No differences in the expression of non-biotinylated cytoplasmic monomeric and dimeric forms of ErbB2 were observed between WT and *ST6GAL1 K.O*. cells. To further explore these observations, we analyzed the dimerization capacity of the remaining ErbB receptor family members, in trastuzumab-treated WT and *ST6GAL1* K.O. cells, by performing protein cross-linking with bis(sulfosuccinimidyl)suberate (BS3) prior to whole cell lysate collection. Interestingly, and in line with the previous result, we observed an increased detection of ErbB2, EGFR and ErbB3 dimeric forms in trastuzumab-treated *ST6GAL1 K.O*. cells, when compared to the WT (Fig. 7C). No significant differences were observed in the detection of ErbB4-containing dimers.

Since ST6Gal1-mediated α2,6-sialylation constitutes a well-established regulator of transmembrane receptor activation, namely by influencing receptor dimerization and membrane turnover rate, we analyzed the phosphorylation levels of 49 cancer-related RTKs in untreated and trastuzumab-treated NCI-N87 WT and *ST6GAL1* K.O. cells with a human phospho-RTK array (Fig. 7D). As previously observed, trastuzumab treatment led to a more severe downregulation of ErbB2 and EGFR phosphorylation in *ST6GAL1* K.O. cells. ErbB3 activation was decreased in *ST6GAL1* K.O. cells regardless of trastuzumab treatment. In the absence of trastuzumab, *ST6GAL1* K.O. cells exhibited increased activation of the Mer RTK. Interestingly, trastuzumab treatment induced the marked phosphorylation of the oncogenic Hepatocyte Growth Factor Receptor (HGF R, c-Met) in *ST6GAL1* K.O. cells. Taken together, these observations support an active role of ST6Gal1-mediated α2,6-sialylation in remodeling the cell surface RTK phosphoproteome of ErbB2-addicted GC cells in response to the treatment with trastuzumab.

## Discussion

Since the diagnosis of GC often takes place after local invasion or distant metastatic dissemination, at which stage tumor resection with curative intent can no longer be performed, current available chemotherapeutic strategies remain ineffective in improving the dismal prognosis of these patients [2]. Furthermore, the high degree of molecular heterogeneity dictating the extremely aggressive behavior of gastric malignant tumors fosters the emergence of both intrinsic and acquired molecular resistance, which, in turn, discourages the use of a “one-size-fits-all” therapeutic approach [22]. Recent multi-omic studies have shown that the genomic, transcriptomic and proteomic profiling of gastric carcinomas allows for the stratification of GC patients into well-defined and clinically relevant subgroups according to the presence of therapeutically targetable molecular alterations, and have further provided the basis for the rational design of clinical trials [23, 24]. The glycomic landscape of gastric neoplasms, on the other hand, remains largely unexplored in the clinical setting. It has only recently been demonstrated how specific glycoforms of oncogenic RTKs actively tune the malignant phenotype of GC cells and further correlate with the clinicopathological features of gastric carcinomas and patient clinical outcome [5–8, 25–27].

Although ErbB2 remains one of the few actionable molecular targets in the GC therapeutic setting, the clinical performance of trastuzumab is largely hampered by the emergence of both intrinsic and acquired molecular resistance. Few studies prior to this one have addressed the functional role of ErbB2 glycosylation in the context of human malignancy [5, 15, 28, 29]. Here, we demonstrate that ErbB2 constitutes a target of both α2,3 and α2,6-sialylation in GC clinical specimens. Future studies encompassing larger patient cohorts are warranted to elucidate the potential of specific ErbB2 glycosylation signatures as robust molecular predictors of GC patient clinical outcome.

The identification of several oncogenic cell surface receptors as molecular targets of ST6Gal1, which is frequently overexpressed in the majority of solid human tumors, has uncovered the active role played by α2,6NeuAc motifs in supporting malignant cell pluripotency, invasive and metastatic capacities, and ability to evade apoptotic pathways and immune-mediated anti-tumor responses [10–14, 30, 31]. However, the role played by ST6Gal1 in the neoplastic transformation of the gastric mucosa remains poorly understood. Having previously identified ErbB2 as a protein target of ST6Gal1-mediated α2,6-sialylation [8], the present study provides a structural and mechanistic basis for the oncogenic role played by ST6Gal1 in trastuzumab resistance, in the context of ErbB2-driven gastric carcinogenesis. The silencing of *ST6GAL1* triggered a drastic remodeling of the cell surface glycosylation profile in ErbB2-dependent GC cells. We postulate that the consequent increase in substrate availability for competing glycosyltransferases, including fucosyltransferases and α2,3-sialyltransferases, underlies the observed changes in glycophenotype. Interestingly, identical alterations were identified in the ErbB2 glycomic landscape in a glycosite-specific manner. Following *ST6GAL1* K.O., the ErbB2 residues N124, N187 and N259 kept their glycan repertoire, solely constituted by oligomannosidic *N*-glycan chains, largely unchanged. However, two glycosylation sites occurring within the receptor’s trastuzumab-binding domain, N530 and N629, underwent a profound reshaping of their terminal glycan motifs, characterized by the enrichment in multi-fucosylated species. Indeed, the glycosylation repertoire of a single glycosylation site is frequently highly heterogeneous, with the relative abundance of structurally distinct glycan structures, or site occupancy, varying significantly, a concept defined as microheterogeneity or site heterogeneity. For these reasons, the comprehensive glycoproteomic mapping of individual proteins, such as the one here performed, is required for the disclosure of context-dependent functional contributions of domain- and site-specific glycan signatures. Indeed, the site-specific glycan composition of prominent cancer-associated proteins can provide valuable insights regarding their oncogenicity, tridimensional structure and binding affinity to therapeutic agents, such as mAbs.

Lastly, α2,6NeuAc-null ErbB2-positive GC cells exhibited a prolonged ErbB2 protein half-life and increased sensitivity to trastuzumab’s cytotoxic effects. These results support a functional role of the cellular- and ErbB2-specific sialylation in the receptor’s cell surface turnover rate and the acquisition of resistance to this therapeutic agent. Moreover, in *ST6GAL1* K.O. cells, an increase in the trastuzumab-induced stabilization of ErbB2-, EGFR- and ErbB3-containing dimers at the cell membrane was observed in parallel with the downregulation of ErbB2 and EGFR intracytoplasmic phosphorylation, which has been previously reported in other epithelial-derived human cancer and nonmalignant in vitro models [14, 32]. These observations highlight how changes in the cellular glycosylation machinery deeply impact RTK dynamics at the cell membrane and downstream signaling. However, whether the observed differential receptor glycosylation patterns directly modulate the non-covalent steric interaction between trastuzumab and ErbB2’s subdomain IV and, consequently, underlie the observed differences in trastuzumab sensitivity requires further investigation.

The treatment of *ST6GAL1* K.O. cells with trastuzumab induced a similar inhibitory effect in the intracellular activation of EGFR, as well as a more pronounced cell surface stabilization of EGFR-containing dimers. Of note, EGFR itself constitutes a molecular target of *N*-linked α2,6-sialylation, which, in turn, has been shown to regulate receptor’s intracellular phosphorylation and dimerization [6, 14]. Moreover, ErbB2 constitutes the preferred dimerization partner of EGFR [33]. It is thus pertinent to postulate that, in the presence of trastuzumab, a downregulation of ErbB2 availability and activation at the cell membrane upon the K.O. of *ST6GAL1* directly affects the receptor’s ability to heterodimerize and activate EGFR. Therefore, following the silencing of *ST6GAL1*, both alterations in the EGFR glycosylation profile and a decrease in ErbB2’s heterodimerization capacity could underlie the observed downregulation in EGFR activation upon trastuzumab treatment. Such observations become relevant when exploiting EGFR inhibition in the personalized therapeutic setting of GC [34]. Furthermore, besides the observed impact in the biology of ErbB receptors, the abrogation of ST6Gal1 expression induced significant alterations in the phosphorylation levels of other cancer-associated RTKs in trastuzumab-treated cells, including the upregulation of c-Met activation, a previously established molecular carrier of α2,6NeuAc motifs [35]. Overall, the herein described molecular mechanism further corroborates the previously reported oncogenic role of ST6Gal1 in other human cancer models [7, 14].

The emergence of molecular resistance to trastuzumab represents a pressing concern hampering the clinical management of GC patients. The future success of personalized therapeutic strategies will certainly rely on the accurate profiling and comprehensive understanding of each tumor’s molecular landscape. Our study provides an additional mechanistic basis further supporting the clinical utility held by the glycosylation status of proteins with critical roles in cancer onset and development [9, 20]. In particular, the clinical implementation of diagnostic tools for the detection of selected ErbB2 glycoforms may become useful in the prediction of trastuzumab therapeutic response, therefore improving GC patient stratification.

## Materials and methods

The Supplementary Materials and Methods document includes detailed procedures for: patient clinical samples and their characterization by immunohistochemistry, lectin histochemistry and in situ PLA analysis (Table S3); selected cell lines, culture conditions and treatments used in all performed functional assays; CRISPR/Cas9 genomic silencing of the *ST6GAL1* gene in NCI-N87 GC cell line, and molecular and phenotypical characterization of the obtained *ST6GAL1* K.O. cell clones (Real-Time quantitative PCR, Western and lectin blot analysis, immunofluorescence [36] and flow cytometry staining); Mass spectrometry (MS)-based characterization ErbB2 glycosylation profile (capillary electrophoresis–electrospray ionization-mass spectrometry (CE–ESI-MS) [37] and Liquid chromatography tandem mass spectrometry (LC–MS/MS)) [38]; functional assays of cell surface glycan labeling, cell proliferation, cell death, metabolic activity, ErbB2 protein half-life, cell surface biotinylation, ErbB dimerization assays, and Human Phospho-Kinase/RTK Arrays (Table S4); Bioinformatic [39, 40] and statistical analysis.

## Supplementary information

Figure S1

Figure S2

Figure S3

Figure S4

Figure S5

Table S1

Table S2

Table S3

Table S4

Supplemental Materials and Methods

Supplementary Figures and Table Legends

## Data Availability

The MS glycoproteomic data have been deposited in the ProteomeXchange Consortium (http://proteomecentral.proteomexchange.org) via the PRIDE partner repository with the dataset identifier PXD017609 [41]. The MS glycomic data have been deposited in the ProteomeXchange Consortium via the MassIVE partner repository with the dataset identifier MSV000084943 (10.25345/C5B38P) [42].
