# Supplementary figures and images for: ST6Gal1 targets the ectodomain of ErbB2 in a site-specific manner and regulates gastric cancer cell sensitivity to trastuzumab
Source: Oncogene. 2021 May 4;40(21):3719–33. doi: 10.1038/s41388-021-01801-w (PMC8154592; doi:10.1038/s41388-021-01801-w)

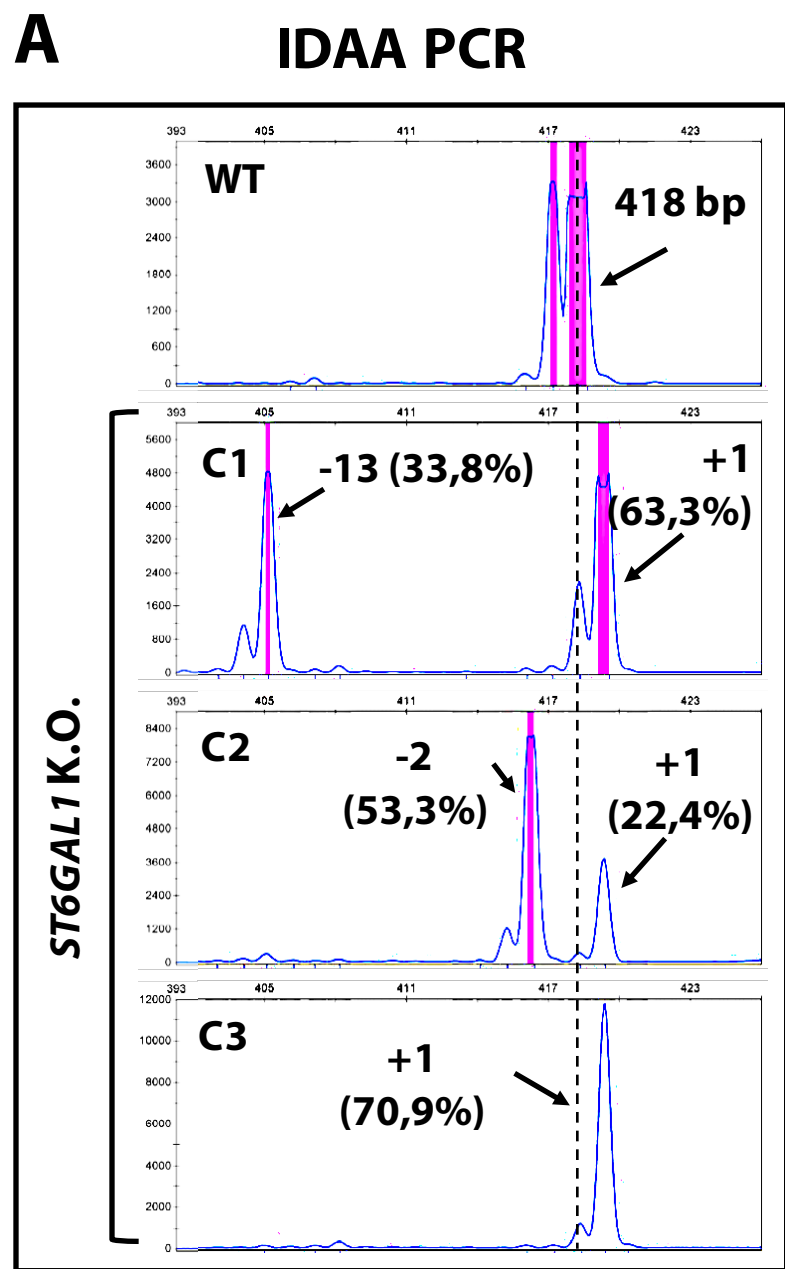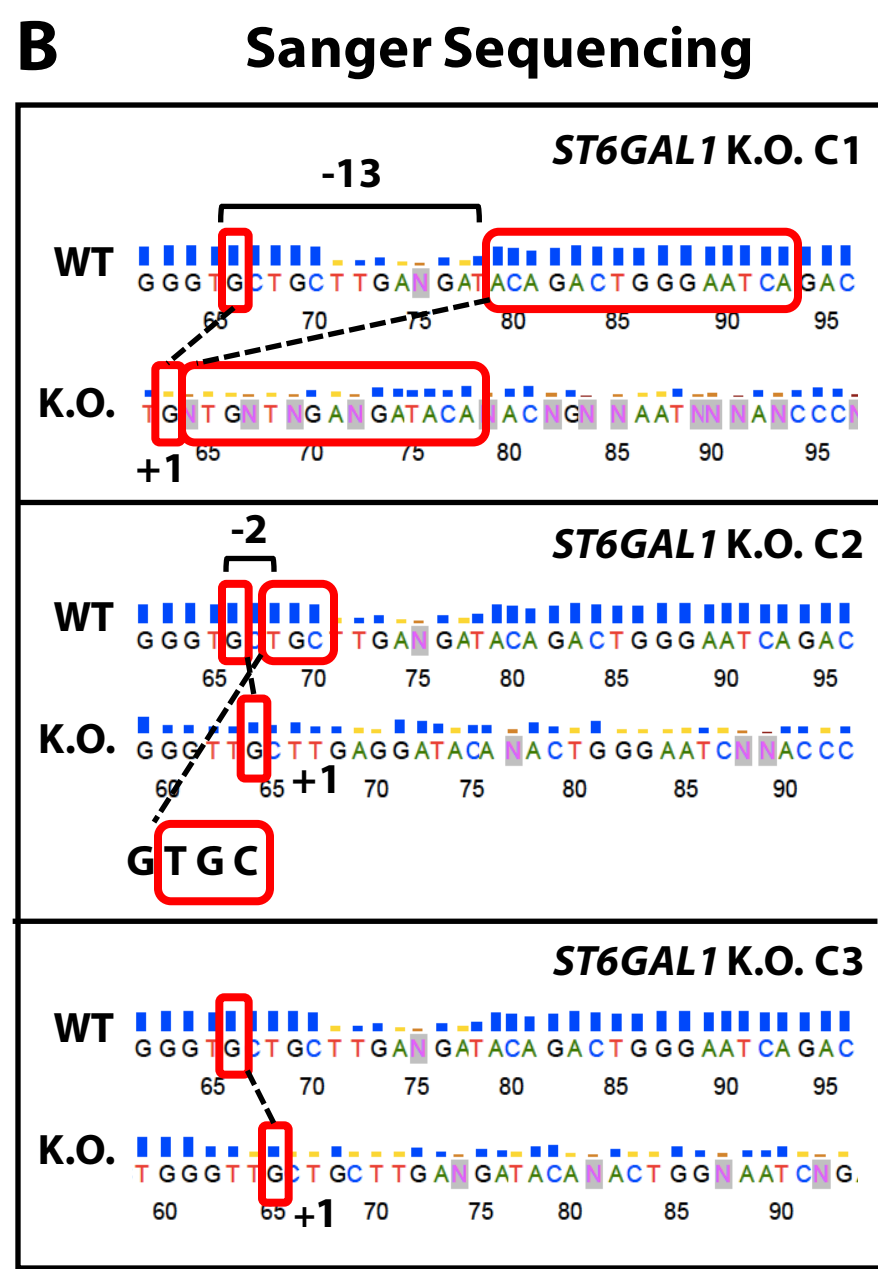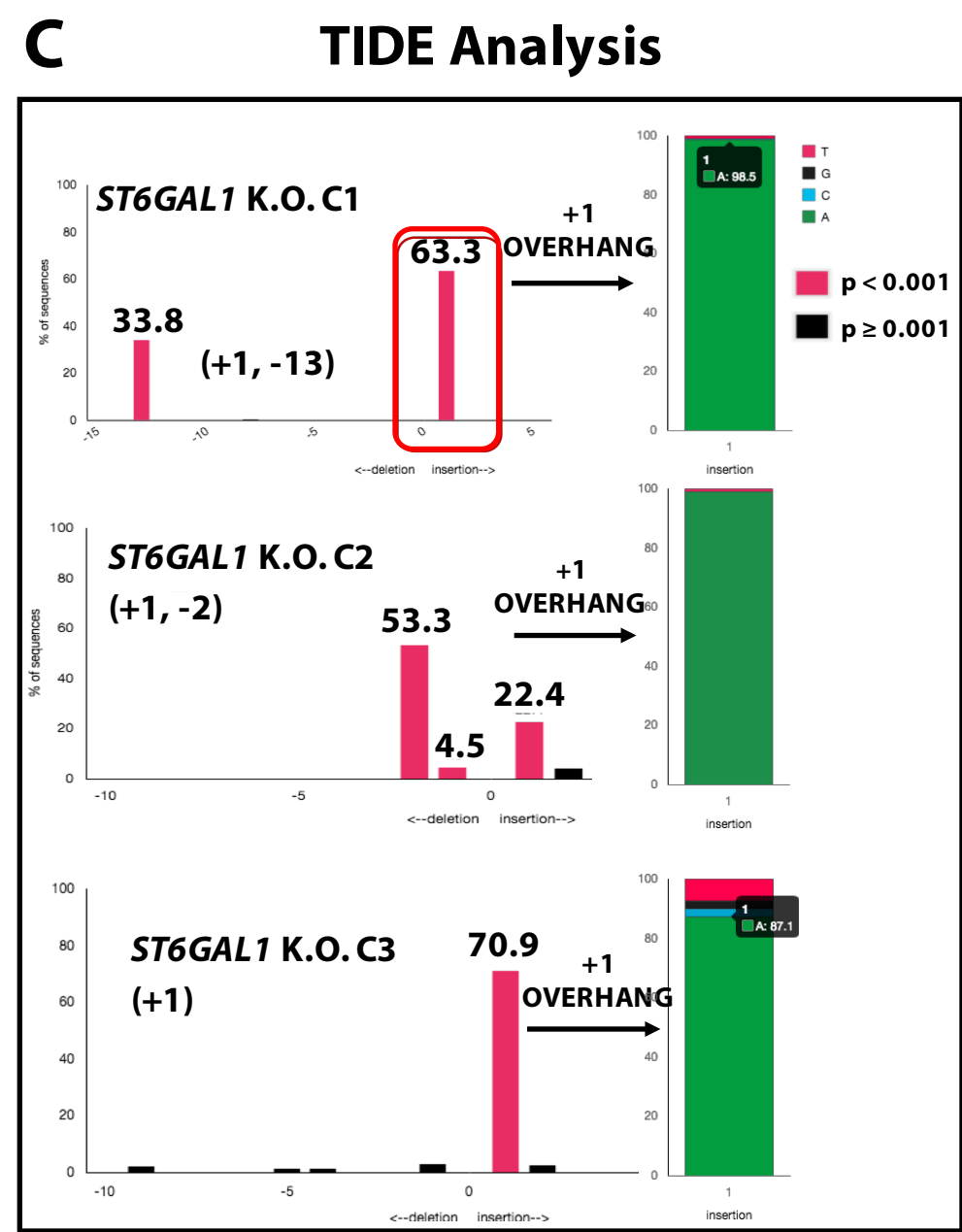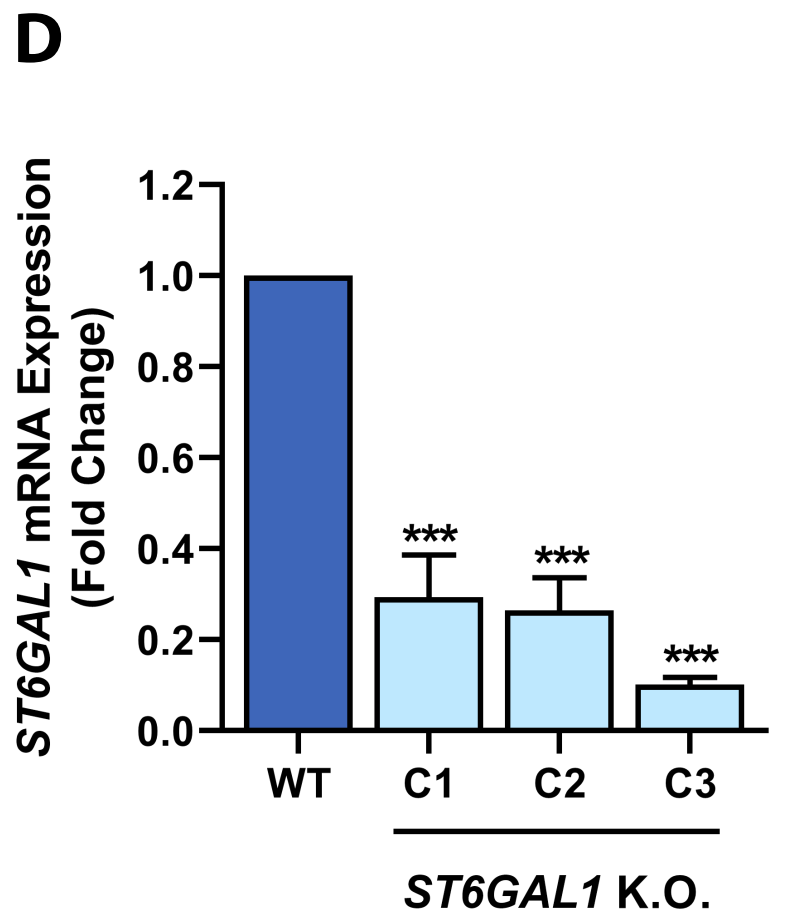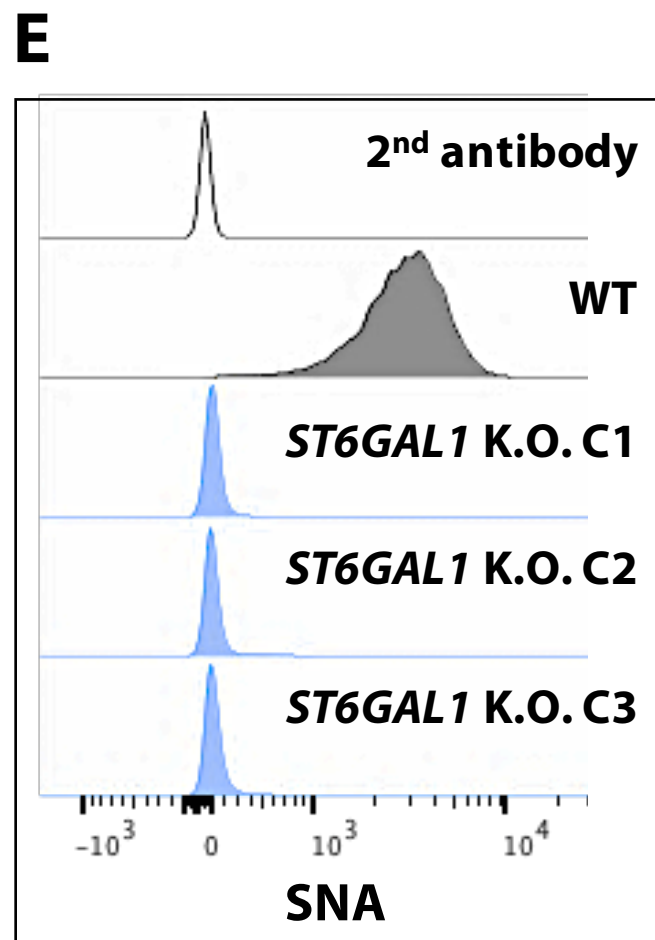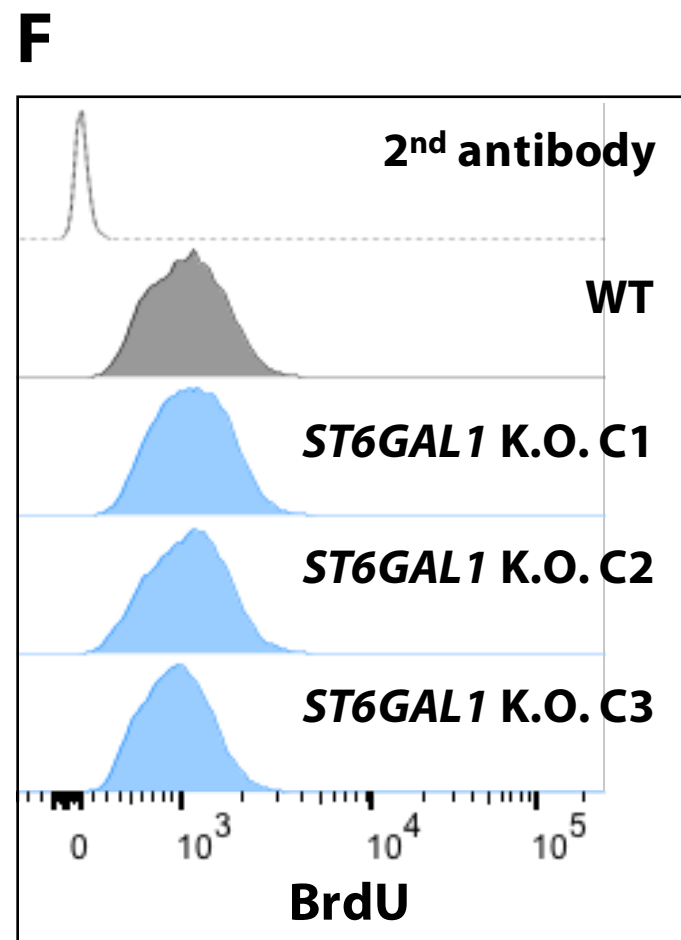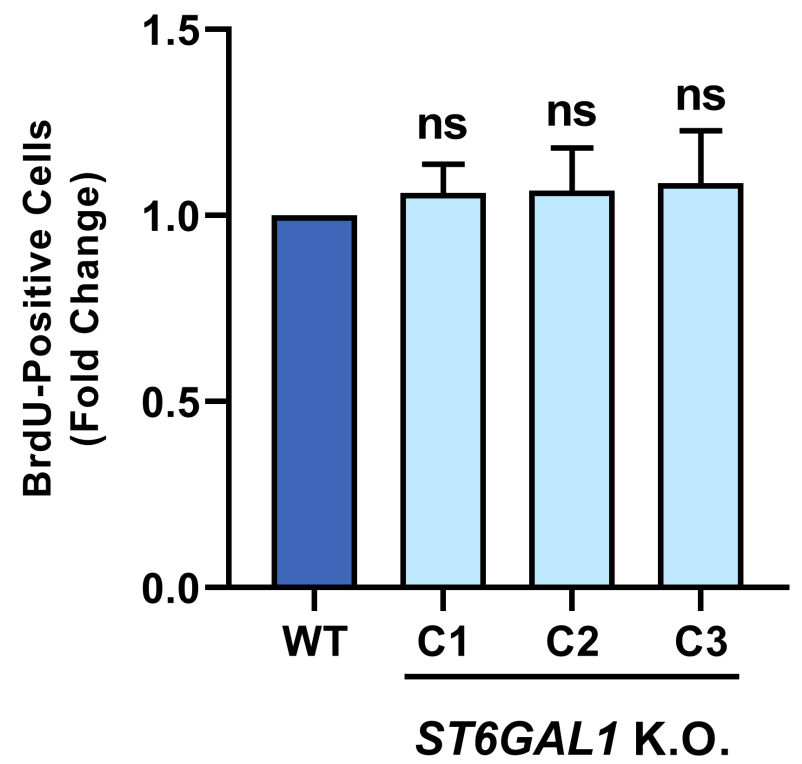

Supplement: Supplementary file 1 — Figure S1 [file 41388_2021_1801_MOESM1_ESM.pdf]

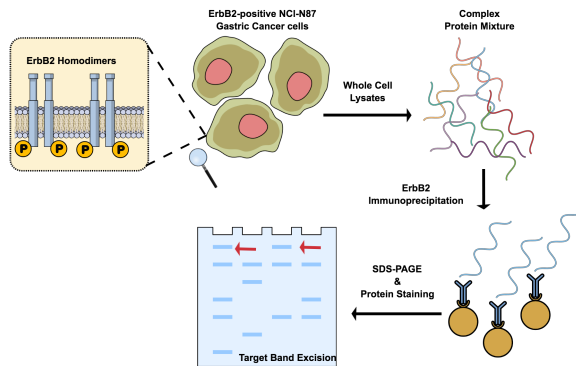

## Glycoproteomic Analysis

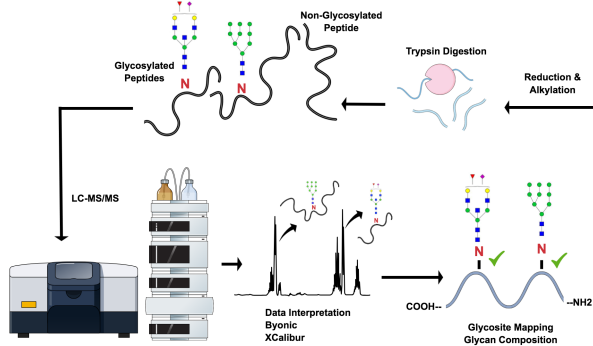

## Glycomic Analysis

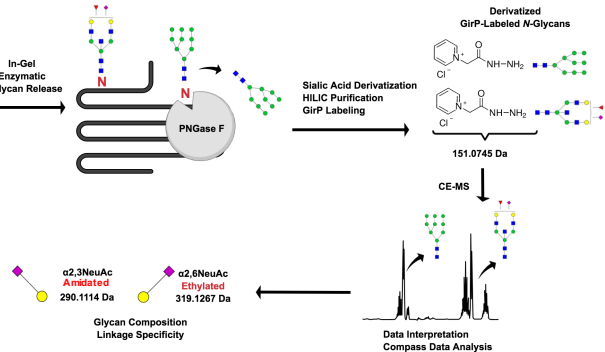

Supplement: Supplementary file 2 — Figure S2 [file 41388_2021_1801_MOESM2_ESM.pdf]

# A

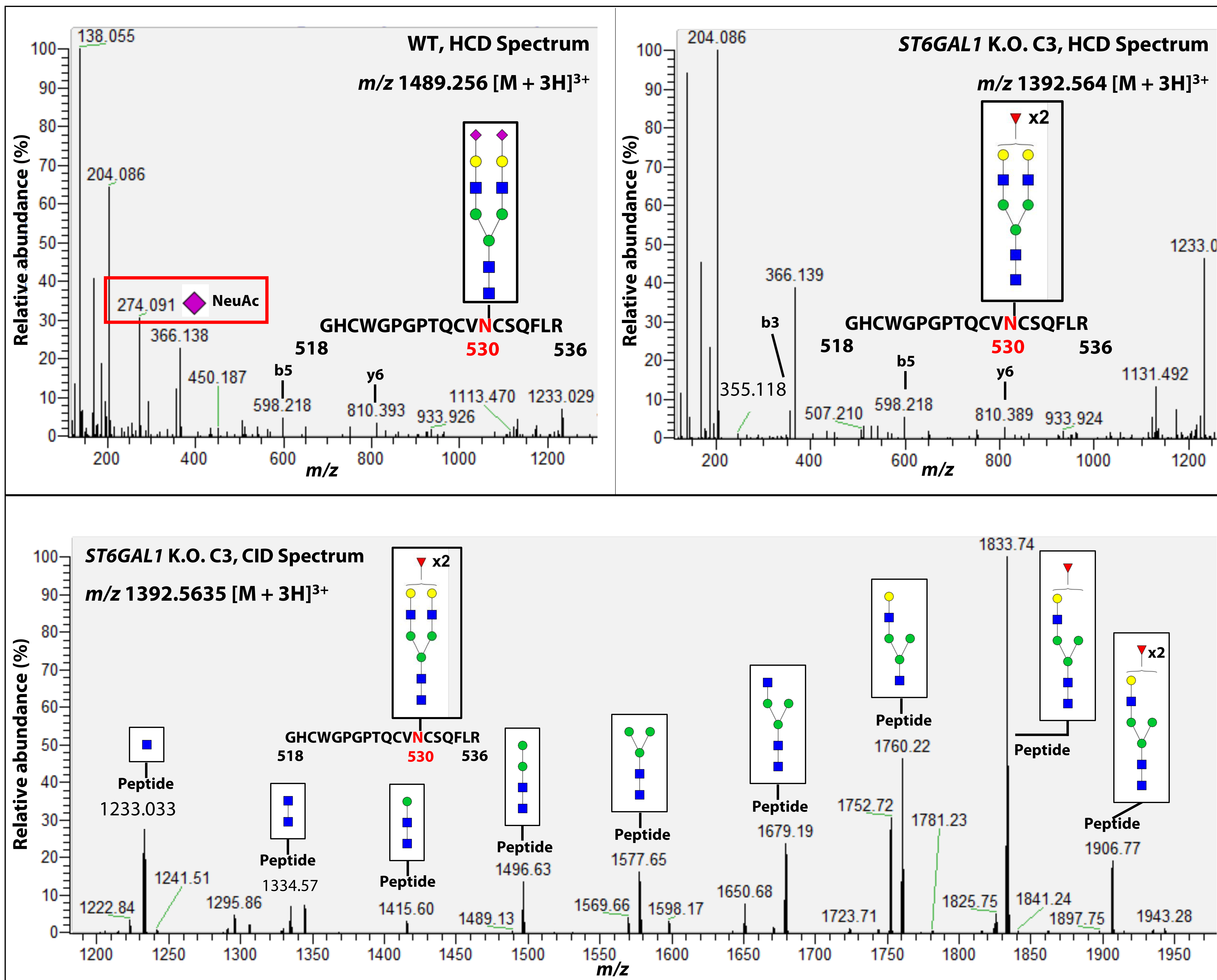

# B

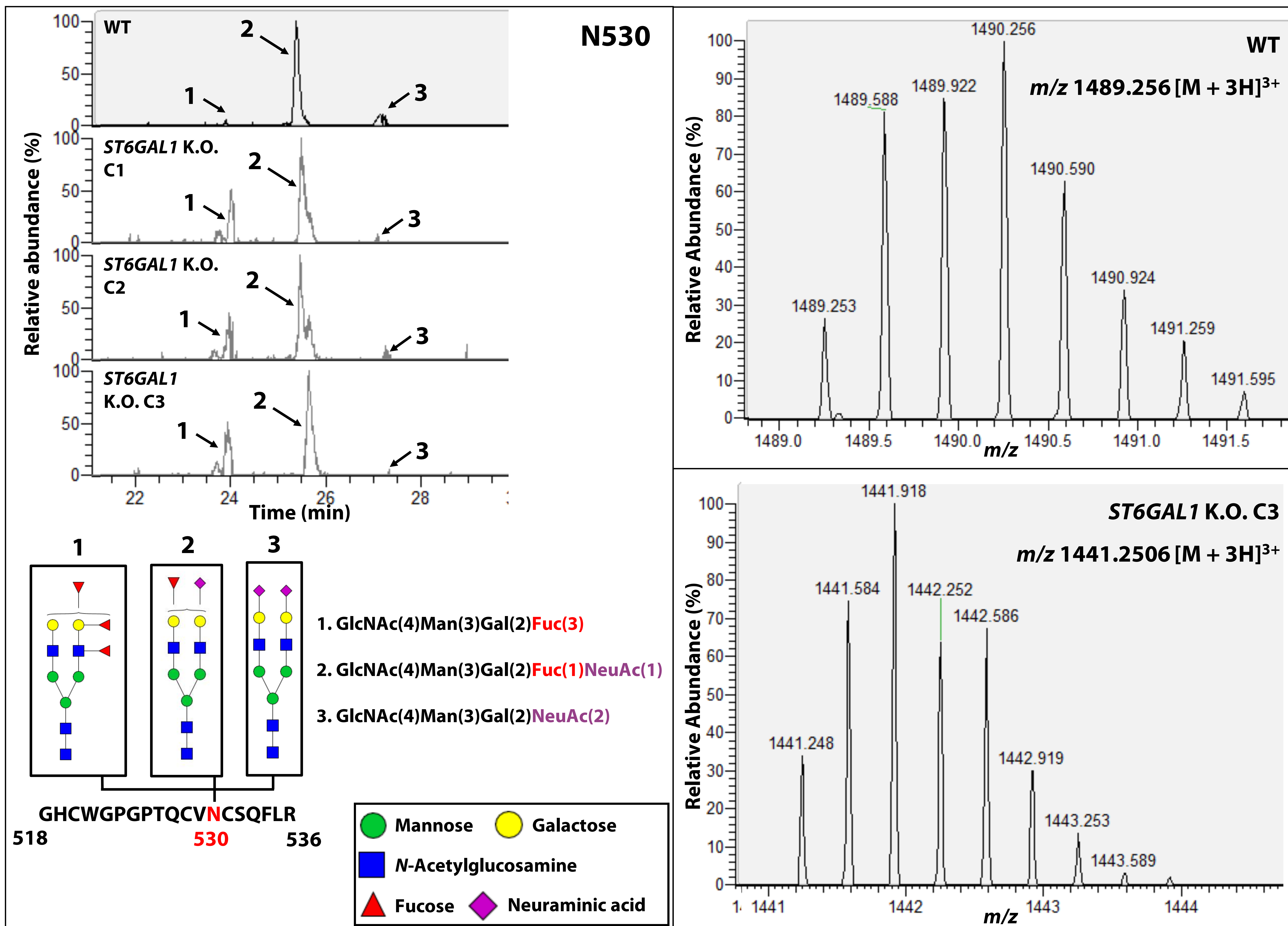

Supplement: Supplementary file 3 — Figure S3 [file 41388_2021_1801_MOESM3_ESM.pdf]

## ErbB2

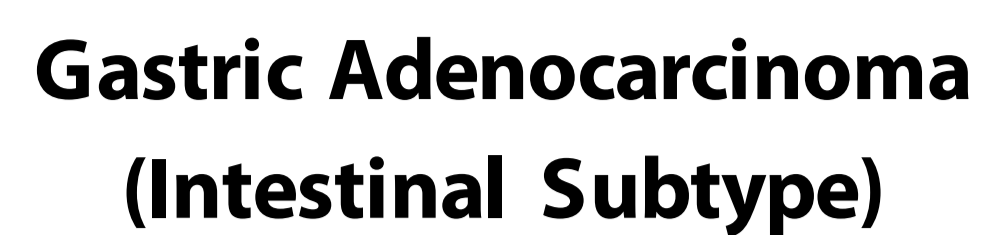

# B

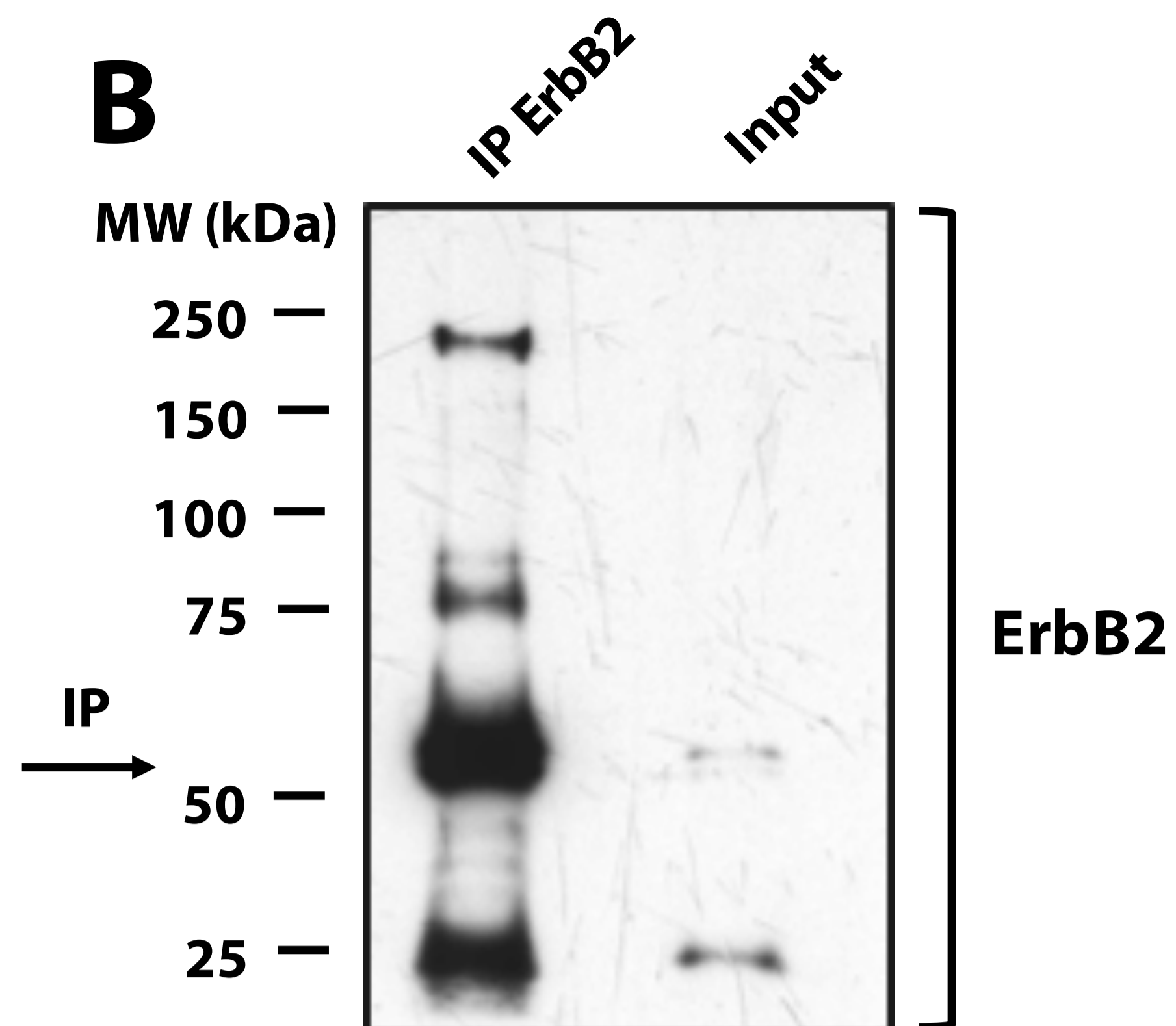

## ErbB2

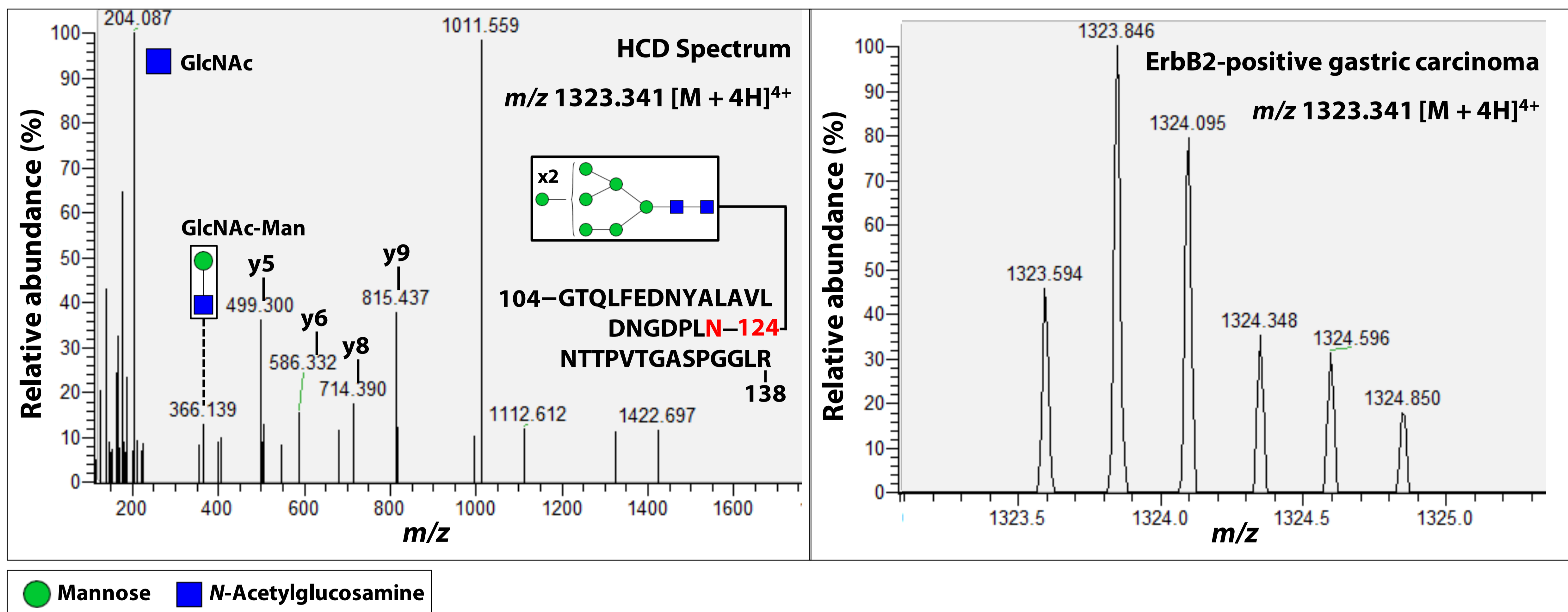

D

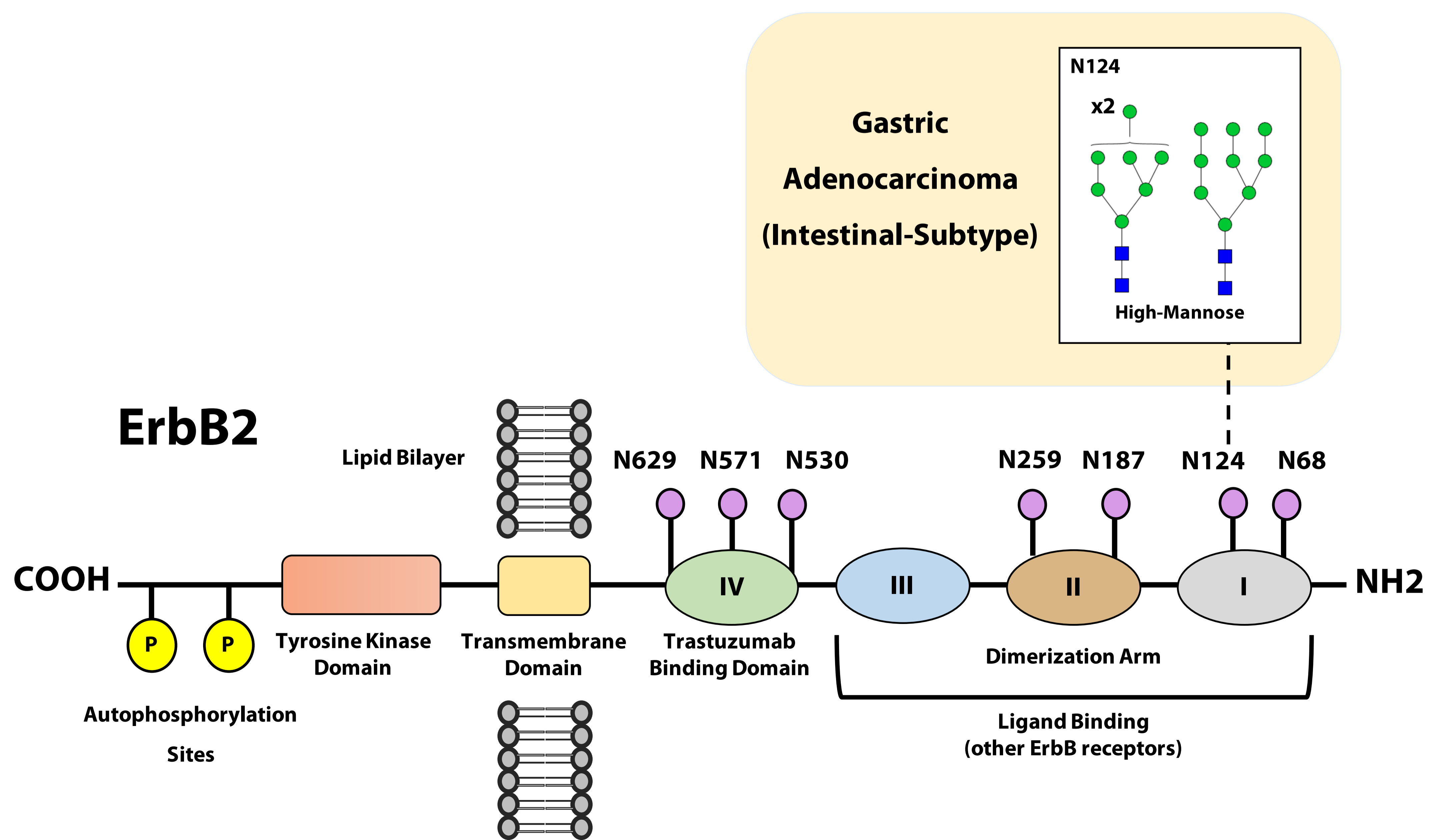

Supplement: Supplementary file 4 — Figure S4 [file 41388_2021_1801_MOESM4_ESM.pdf]

**A**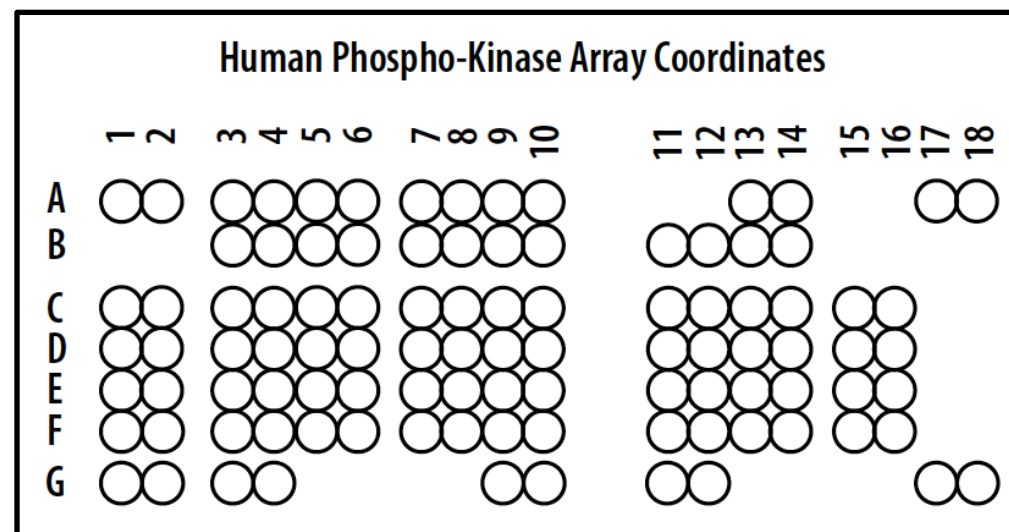**B**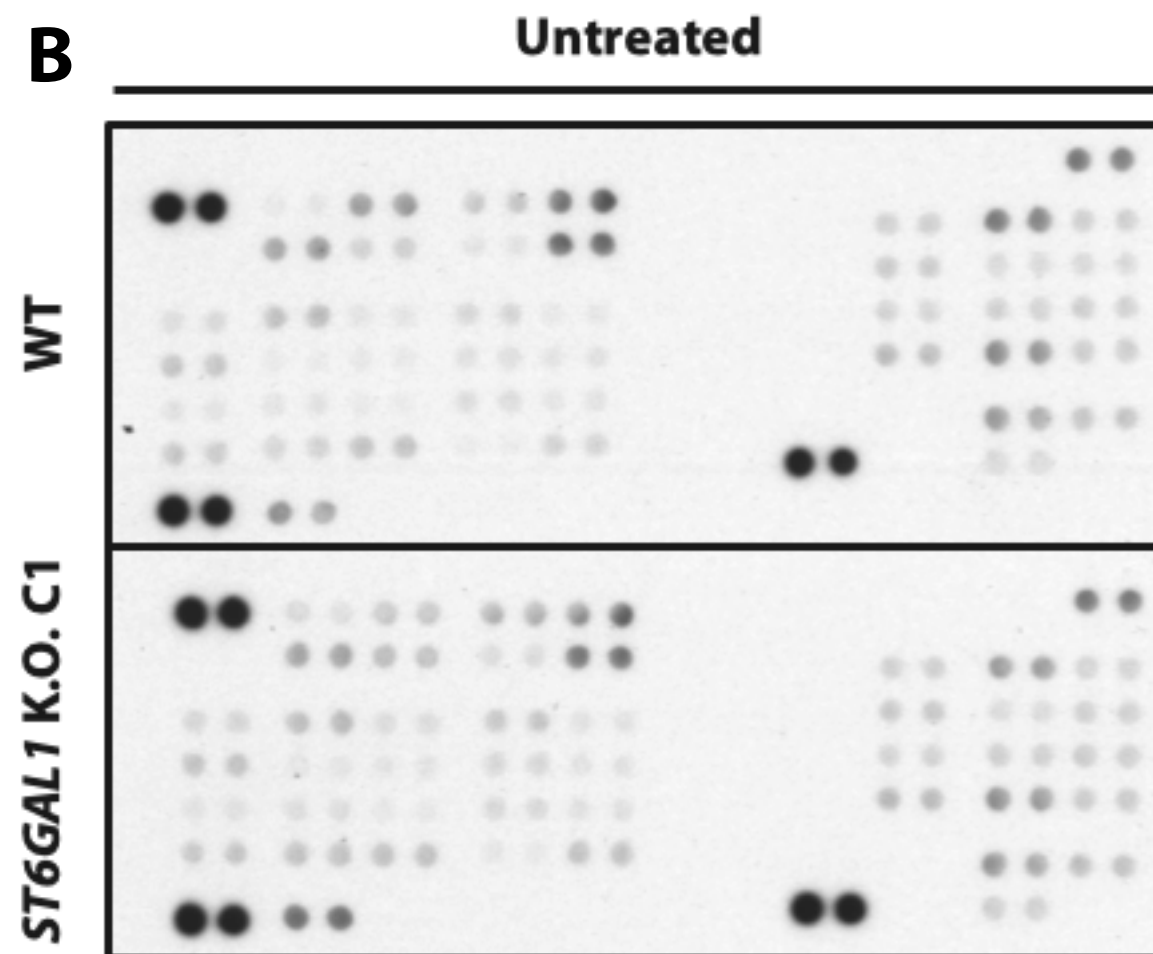**C**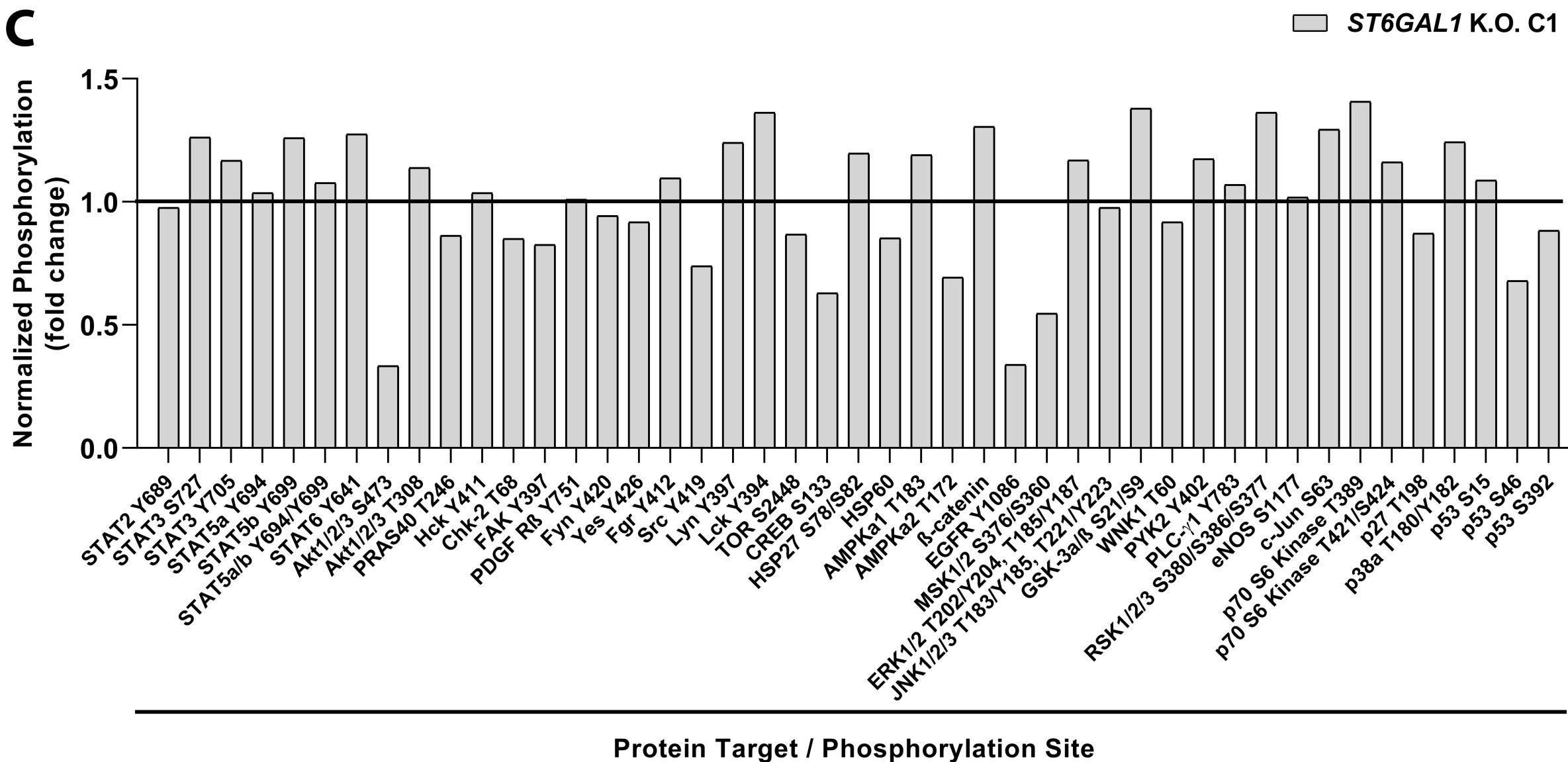

Supplement: Supplementary file 5 — Figure S5 [file 41388_2021_1801_MOESM5_ESM.pdf]
