# Supplementary material for: ST6Gal1 targets the ectodomain of ErbB2 in a site-specific manner and regulates gastric cancer cell sensitivity to trastuzumab
Source: Oncogene. 2021 May 4;40(21):3719–33. doi: 10.1038/s41388-021-01801-w (PMC8154592; doi:10.1038/s41388-021-01801-w)
Supplement: Supplementary file 6 — Table S1 [file 41388_2021_1801_MOESM6_ESM.docx]

**Table S1.** Clinicopathological data and glycan biomarker expression in ErbB2-positive gastric cancer clinical samples.

|  |  | **BIOMARKER** | | | | | | | | | | | |
| --- | --- | --- | --- | --- | --- | --- | --- | --- | --- | --- | --- | --- | --- |
|  |  | **SLe^a^** | | ***p*-value** | **PLA ErbB2/SLe^a^** | | ***p*-value** | **SNA** | | ***p*-value** | **PLA ErbB2/SNA** | | ***p*-value** |
|  |  | **Pos.** | **Neg.** |  | **Pos.** | **Neg.** |  | **Pos.** | **Neg.** |  | **Pos.** | **Neg.** |  |
| **Stage (pTNM)** | **I (n=1)** | 0 | 1 | 0.478 | 1 | 0 | 0.459 | 0 | 1 | n.a. | 1 | 0 | 0.091 |
|  | **II (n=1)** | 0 | 1 |  | 0 | 1 |  | 0 | 1 |  | 1 | 0 |  |
|  | **III (n=8)** | 0 | 8 |  | 4 | 4 |  | 0 | 8 |  | 2 | 6 |  |
|  | **IV (n=9)** | 2 | 7 |  | 6 | 3 |  | 0 | 9 |  | 7 | 2 |  |
|  | **Total** | 2 | 17 |  | 11 | 8 |  | 0 | 19 |  | 11 | 8 |  |
| **Size / Extension (T)** | **T1 (n=0)** | 0 | 0 | 0.063 | 0 | 0 | 0.312 | 0 | 0 | n.a. | 0 | 0 | 0.312 |
|  | **T2 (n=4)** | 1 | 3 |  | 3 | 1 |  | 0 | 4 |  | 3 | 1 |  |
|  | **T3 (n=13)** | 0 | 13 |  | 6 | 7 |  | 0 | 13 |  | 6 | 7 |  |
|  | **T4 (n=0)** | 0 | 0 |  | 0 | 0 |  | 0 | 0 |  | 0 | 0 |  |
|  | **Undetermined (n=2)** | 1 | 1 |  | 2 | 0 |  | 0 | 2 |  | 2 | 0 |  |
|  | **Total** | 2 | 17 |  | 11 | 8 |  | 0 | 19 |  | 11 | 8 |  |
| **Lymph Node Metastasis (N)** | **N0 (n=1)** | 0 | 1 | 0.202 | 1 | 0 | 0.694 | 0 | 1 | n.a. | 1 | 0 | 0.642 |
|  | **N1 (n=3)** | 1 | 2 |  | 2 | 1 |  | 0 | 3 |  | 2 | 1 |  |
|  | **N2 (n=5)** | 0 | 5 |  | 2 | 3 |  | 0 | 5 |  | 2 | 3 |  |
|  | **N3 (n=7)** | 0 | 7 |  | 4 | 3 |  | 0 | 7 |  | 3 | 4 |  |
|  | **Undetermined (n=3)** | 1 | 2 |  | 2 | 1 |  | 0 | 3 |  | 3 | 0 |  |
|  | **Total** | 2 | 17 |  | 11 | 8 |  | 0 | 19 |  | 11 | 8 |  |
| **Distant Metastasis (M)** | **M0 (n=7)** | 0 | 7 | 0.155 | 3 | 4 | 0.447 | 0 | 7 | n.a. | 3 | 4 | 0.067 |
|  | **M1 (n=8)** | 2 | 6 |  | 5 | 3 |  | 0 | 8 |  | 7 | 1 |  |
|  | **Undetermined (n=4)** | 0 | 4 |  | 3 | 1 |  | 0 | 4 |  | 1 | 3 |  |
|  | **Total** | 2 | 17 |  | 11 | 8 |  | 0 | 19 |  | 11 | 8 |  |
| **Overall Survival (OS)** | **Median (months ± SD)** | 18.5 ± 24.1 | | | | | | | | | | | |
|  | **Mean**  **(months ± SD)** | 29.44 ± 24.08 | | | | | | | | | | | |
| **Age** | | 61.47 ± 10.79 | | | | | | | | | | | |
| **Gender** | **Female** | 7 | | | | | | | | | | | |
|  | **Male** | 12 | | | | | | | | | | | |
