# Supplementary material for: ST6Gal1 targets the ectodomain of ErbB2 in a site-specific manner and regulates gastric cancer cell sensitivity to trastuzumab
Source: Oncogene. 2021 May 4;40(21):3719–33. doi: 10.1038/s41388-021-01801-w (PMC8154592; doi:10.1038/s41388-021-01801-w)
Supplement: Supplementary file 8 — Table S3 [file 41388_2021_1801_MOESM8_ESM.docx]

**Table S3.** Specification of primary antibodies and lectins.

| **Antibody / Lectin** | **Clone** | **Source** | **Catalog Number** | **Technique** | **Dilution** |
| --- | --- | --- | --- | --- | --- |
| **ErbB2** | SP3 | Thermo Fisher Scientific | MA5-14509 | IHC, PLA | 1:80 |
|  | 29D8 | Cell Signaling Technology | 2165S | IF, IP, WB | 1:200, 1:100, 1:1000 |
| **SLe^a^** | CA 19.9 | Santa Cruz Biotechnology | sc-59481 | IHC, PLA, FC | 1:500, 1:500, 1:100 |
| **ST6Gal1** | - | R&D Systems | AF5294 | IHC, IF, WB | 1:40, 1:200, 1:500 |
| **STn** | B72.3 | [36] | - | IF | 1:5 |
| **α-Tubulin** | DM1A | Sigma-Aldrich |  | WB | 1:10 000 |
| **SNA** | - | Vector Laboratories | B-1305-2 | IHC, PLA, IF, WB | 1:1000, 1:1000, 1:500, 1:3000 |
| **SNA-FITC** |  |  | FL-1301-2 | FC | 1:2000 |
| **MAL-II** |  |  | B-1265-1 | FC | 1:100 |
| **AAL** |  |  | B-1395-1 | FC | 1:100 |
| **LTL** |  |  | B-1325-2 | FC | 1:200 |
| **UEA-I** |  |  | B-1065-2 | FC, WB | 1:200, 1:2000 |
| **Phosphorylated ErbB2 (Tyr1221/1222)** | - | Cell Signaling Technology | 2249 | WB | 1:1000 |
| **EGFR** | D38B1 | Cell Signaling Technology | 4267 | WB | 1:1000 |
| **Phosphorylated EGFR (Y1086)** | - | Thermo Fisher Scientific | 39-9700 | WB | 1:2000 |
| **ErbB3** | D22C5 | Cell Signaling Technology | 12708S | WB | 1:1000 |
| **ErbB4** | 111B2 | Cell Signaling Technology | 4795S | WB | 1:1000 |
| **Cytochrome c** | 7H8 | Santa Cruz Biotechnology | sc-13560 | WB | 1:200 |
| **β-Actin** | 13E5 | Cell Signaling Technology | 4970S | WB | 1:1000 |
