# Supplementary material for: ST6Gal1 targets the ectodomain of ErbB2 in a site-specific manner and regulates gastric cancer cell sensitivity to trastuzumab
Source: Oncogene. 2021 May 4;40(21):3719–33. doi: 10.1038/s41388-021-01801-w (PMC8154592; doi:10.1038/s41388-021-01801-w)
Supplement: Supplementary file 9 — Table S4 [file 41388_2021_1801_MOESM9_ESM.docx]

**Table S4.** Human Phospho-Kinase/RTK Array Membrane Coordinates.

| **Proteome Profiler Human Phospho-Kinase Array (ARY003B)** | | | |
| --- | --- | --- | --- |
| **Membrane** | **Protein Target** | **Phosphorylation Site** | **Coordinate** |
| **A** | **Reference Spot** | **-** | **G1** |
|  |  |  | **G2** |
|  | **PRAS40** | **T246** | **G3** |
|  |  |  | **G4** |
|  | **Hck** | **Y411** | **F1** |
|  |  |  | **F2** |
|  | **Chk-2** | **T68** | **F3** |
|  |  |  | **F4** |
|  | **FAK** | **Y397** | **F5** |
|  |  |  | **F6** |
|  | **PDGF Rβ** | **Y751** | **F7** |
|  |  |  | **F8** |
|  | **STAT5a/b** | **Y694/Y699** | **F9** |
|  |  |  | **F10** |
|  | **Fyn** | **Y420** | **E1** |
|  |  |  | **E2** |
|  | **Yes** | **Y426** | **E3** |
|  |  |  | **E4** |
|  | **Fgr** | **Y412** | **E5** |
|  |  |  | **E6** |
|  | **STAT6** | **Y641** | **E7** |
|  |  |  | **E8** |
|  | **STAT5b** | **Y699** | **E9** |
|  |  |  | **E10** |
|  | **Src** | **Y419** | **D1** |
|  |  |  | **D2** |
|  | **Lyn** | **Y397** | **D3** |
|  |  |  | **D4** |
|  | **Lck** | **Y394** | **D5** |
|  |  |  | **D6** |
|  | **STAT2** | **Y689** | **D7** |
|  |  |  | **D8** |
|  | **STAT5a** | **Y694** | **D9** |
|  |  |  | **D10** |
|  | **TOR** | **S2448** | **C1** |
|  |  |  | **C2** |
|  | **CREB** | **S133** | **C3** |
|  |  |  | **C4** |
|  | **HSP27** | **S78/S82** | **C5** |
|  |  |  | **C6** |
|  | **AMPKα2** | **T172** | **C7** |
|  |  |  | **C8** |
|  | **β-catenin** | **-** | **C9** |
|  |  |  | **C10** |
|  | **EGFR** | **Y1086** | **B3** |
|  |  |  | **B4** |
|  | **MSK1/2** | **S376/S360** | **B5** |
|  |  |  | **B6** |
|  | **AMPKa1** | **T183** | **B7** |
|  |  |  | **B8** |
|  | **Akt 1/2/3** | **S473** | **B9** |
|  |  |  | **B10** |
|  | **Reference Spot** | **-** | **A1** |
|  |  |  | **A2** |
|  | **p38α** | **T180/Y182** | **A3** |
|  |  |  | **A4** |
|  | **ERK1/2** | **T202/Y204, T185/Y187** | **A5** |
|  |  |  | **A6** |
|  | **JNK 1/2/3** | **T183/Y185, T221/Y223** | **A7** |
|  |  |  | **A8** |
|  | **GSK-3α/β** | **S21/S9** | **A9** |
|  |  |  | **A10** |
| **B** | **HSP60** | **-** | **G11** |
|  |  |  | **G12** |
|  | **STAT3** | **S727** | **F11** |
|  |  |  | **F12** |
|  | **WNK1** | **T60** | **F13** |
|  |  |  | **F14** |
|  | **PYK2** | **Y402** | **F15** |
|  |  |  | **F16** |
|  | **STAT3** | **Y705** | **E11** |
|  |  |  | **E12** |
|  | **p27** | **T198** | **E13** |
|  |  |  | **E14** |
|  | **PLC-𝛄1** | **Y783** | **E15** |
|  |  |  | **E16** |
|  | **p70 S6 Kinase** | **T421/S424** | **D11** |
|  |  |  | **D12** |
|  | **RSK1/2/3** | **S380/S386/S377** | **D13** |
|  |  |  | **D14** |
|  | **eNOS** | **S1177** | **D15** |
|  |  |  | **D16** |
|  | **p70 S6 Kinase** | **T389** | **C11** |
|  |  |  | **C12** |
|  | **p53** | **S15** | **C13** |
|  |  |  | **C14** |
|  | **c-Jun** | **S63** | **C15** |
|  |  |  | **C16** |
|  | **Akt1/2/3** | **T308** | **B11** |
|  |  |  | **B12** |
|  | **p53** | **S46** | **B13** |
|  |  |  | **B14** |
|  | **p53** | **S392** | **A13** |
|  |  |  | **A14** |
|  | **Reference Spot** | **-** | **A17** |
|  |  |  | **A18** |

| **Proteome Profiler Human Phospho-RTK Array (ARY001B)** | | |
| --- | --- | --- |
| **Membrane** | **Protein Target** | **Coordinate** |
| **-** | **Reference Spot** | **A1** |
|  |  | **A2** |
|  | **Reference Spot** | **A23** |
|  |  | **A24** |
|  | **EGFR** | **B1** |
|  |  | **B2** |
|  | **ErbB2** | **B3** |
|  |  | **B4** |
|  | **ErbB3** | **B5** |
|  |  | **B6** |
|  | **ErbB4** | **B7** |
|  |  | **B8** |
|  | **FGFR1** | **B9** |
|  |  | **B10** |
|  | **FGFR2a** | **B11** |
|  |  | **B12** |
|  | **FGFR3** | **B13** |
|  |  | **B14** |
|  | **FGFR4** | **B15** |
|  |  | **B16** |
|  | **Insulin R** | **B17** |
|  |  | **B18** |
|  | **IGF-1R** | **B19** |
|  |  | **B20** |
|  | **Axl** | **B21** |
|  |  | **B22** |
|  | **Dtk** | **B23** |
|  |  | **B24** |
|  | **Mer** | **C1** |
|  |  | **C2** |
|  | **HGFR (c-Met)** | **C3** |
|  |  | **C4** |
|  | **MSPR** | **C5** |
|  |  | **C6** |
|  | **PDGFRɑ** | **C7** |
|  |  | **C8** |
|  | **PDGFRβ** | **C9** |
|  |  | **C10** |
|  | **SCFR** | **C11** |
|  |  | **C12** |
|  | **Flt-3** | **C13** |
|  |  | **C14** |
|  | **MCSFR** | **C15** |
|  |  | **C16** |
|  | **c-Ret** | **C17** |
|  |  | **C18** |
|  | **ROR** | **C19** |
|  |  | **C20** |
|  | **ROR2** | **C21** |
|  |  | **C22** |
|  | **Tie-1** | **C23** |
|  |  | **C24** |
|  | **Tie-2** | **D1** |
|  |  | **D2** |
|  | **Trk** | **D3** |
|  |  | **D4** |
|  | **TrkB** | **D5** |
|  |  | **D6** |
|  | **TrkC** | **D7** |
|  |  | **D8** |
|  | **VEGFR** | **D9** |
|  |  | **D10** |
|  | **VEGFR2** | **D11** |
|  |  | **D12** |
|  | **VEGFR3** | **D13** |
|  |  | **D14** |
|  | **MuSK** | **D15** |
|  |  | **D16** |
|  | **EphA1** | **D17** |
|  |  | **D18** |
|  | **EphA2** | **D19** |
|  |  | **D20** |
|  | **Eph3** | **D21** |
|  |  | **D22** |
|  | **Eph4** | **D23** |
|  |  | **D24** |
|  | **EphA6** | **E1** |
|  |  | **E2** |
|  | **EphA7** | **E3** |
|  |  | **E4** |
|  | **EphB1** | **E5** |
|  |  | **E6** |
|  | **EphB2** | **E7** |
|  |  | **E8** |
|  | **EphB4** | **E9** |
|  |  | **E10** |
|  | **EphB6** | **E11** |
|  |  | **E12** |
|  | **ALK** | **E13** |
|  |  | **E14** |
|  | **DDR1** | **E15** |
|  |  | **E16** |
|  | **DDR2** | **E17** |
|  |  | **E18** |
|  | **EphAA5** | **E19** |
|  |  | **E20** |
|  | **EphA10** | **E21** |
|  |  | **E22** |
|  | **Reference Spot** | **F1** |
|  |  | **F2** |
|  | **EphB3** | **F5** |
|  |  | **F6** |
|  | **RYK** | **F7** |
|  |  | **F8** |
|  | **Control (-)** | **F23** |
|  |  | **F24** |
