# Supplementary material for: ST6Gal1 targets the ectodomain of ErbB2 in a site-specific manner and regulates gastric cancer cell sensitivity to trastuzumab
Source: Oncogene. 2021 May 4;40(21):3719–33. doi: 10.1038/s41388-021-01801-w (PMC8154592; doi:10.1038/s41388-021-01801-w)
Supplement: Supplementary file 10 — Supplemental Materials and Methods [file 41388_2021_1801_MOESM10_ESM.docx]

**Supplemental Materials and Methods**

**Patients and clinical samples**

A retrospective series of 173 primary gastric carcinoma specimens was obtained from the archives of the Portuguese Institute of Oncology (IPO) hospital, Porto, Portugal. Human tumor sample collection and handling were performed in accordance with the national regulative law for the handling of biological specimens from tumor banks, and the international Helsinki declaration, following patient's written informed consent, previously approved by the local institutional Ethical committee. All clinicopathological information was obtained from patients' clinical records. This series, collected from 2005 to 2015, comprised both intestinal- ad diffuse-subtype gastric carcinomas, according to Lauren’s histopathological classification. The clinicopathological data of patients harboring ErbB2-positive tumors (n=19), which were selected for further *in situ* Proximity Ligation Assay (PLA) analysis, are depicted in Table S1. For immunohistochemistry and PLA analysis, formalin-fixed paraffin-embedded (FFPE) tissue sections were used. For total protein extraction and subsequent ErbB2 immunoprecipitation, we used fresh frozen tissue samples, preserved at -80ºC.

**Primary antibodies and lectins**

All primary antibodies and lectins used in this study, as well as their working conditions, are listed in Table S3.

**Immuno- and lectin histochemistry, and *in situ* Proximity Ligation Assay**

The expression of ErbB2, sialyl Lewis a (SLe^a^) and α2,6-linked sialic acids (α2,6NeuAc), recognized by the *Sambucus nigra* agglutinin (SNA), was analyzed in a series of FFPE primary gastric carcinomas. Briefly, deparaffinization and rehydration of FFPE tissue sections was performed, followed by heat-induced antigen retrieval with citrate buffer (10 mM, pH = 6.0), and neutralization of endogenous peroxidase activity with 3% H_2_O_2_. For blocking of non-specific background staining, the following conditions were used: ErbB2 – UltraVision Protein Block (Thermo Fisher Scientific, Waltham, MA, USA), 15 min room temperature (RT); SLe^a^ – Normal rabbit serum (Dako, Berlin, Germany) 1:5 in 10% bovine serum albumin (BSA) 30 min RT; SNA – 10% BSA 30 min RT. Tissue sections were incubated for 2h at RT with primary antibodies and lectins (Table S3), followed by: ErbB2 – horseradish peroxidase-labeled polymer (Dako) 30 min at RT; SLe^a^ – rabbit anti-mouse biotin-labeled secondary antibody (Dako) 30 min at RT; SLe^a^ and SNA – Vectastain ABC kit (Vector Laboratories, Burlingame, CA, USA) 30 min at RT. Tissue sections underwent chromogenic staining with DAB (Sigma-Aldrich, St. Louis, MO, USA) supplemented with 0.01% H_2_O_2_, and nuclear staining was performed with Mayers' hematoxylin.

Molecular proximity between ErbB2 and SLe^a^ or SNA (α2,6NeuAc) was determined by *in situ* PLA staining of ErbB2-positive gastric carcinoma FFPE tissue sections, using the Duolink^TM^ In Situ PLA technology (Sigma-Aldrich), according to manufacturer’s instructions. Briefly, tissue slides were subject to deparaffinization, rehydration, antigen retrieval and blocked. Then, tissue sections were simultaneously incubated with primary antibodies or lectins, as described in Table S3, for 2h at RT. Tissue slides were incubated with DuoLink^TM^ PLA probes: anti-rabbit minus 1:5 (anti-primary ErbB2 antibody), anti-mouse plus 1:5 (anti-primary SLe^a^ antibody) (Sigma-Aldrich) and streptavidin plus 1:80 (anti-biotinylated SNA) for 1h at 37ºC. The remaining steps were performed according to the standard manufacturer’s protocol.

The immunohistochemical staining of each individual antigen was evaluated by a pathologist using a semi-quantitative method: the percentage of stained cells and signal intensity score (0-3). For PLA staining, only the percentage of stained cells was determined. Images were acquired under 200x and 630x (insert) magnifications with a Zeiss Optical Microscope (Zeiss, Oberkochen, Germany).

**Cell culture and treatments**

The intestinal-subtype gastric cancer (GC) cell lines NCI-N87 and MKN-74 were purchased from the American Type Culture Collection (Manassas, VA, USA). The diffuse-subtype GC MKN45 cell line was obtained from the Japanese Cancer Research Bank (Tsukuba, Japan), and had been previously stably transfected with the full-length human *ST6GALNAC1* gene [16]. All cell lines were grown in monolayer in uncoated cell culture flasks, at 37ºC in an atmosphere of 5% CO_2_. NCI-N87, MKN-74 and MKN45 ST6GalNAc1 cell lines were cultured in RPMI 1640 GlutaMAX, HEPES medium (Gibco, Waltham, MA, USA). MKN45 ST6GalNAc1 culture medium was further supplemented with 0.5 mg/mL of G418 selection antibiotic (Invivogen, San Diego, CA, USA). All culture media were supplemented with 10% heat-inactivated fetal bovine serum (FBS) (Biowest, Riverside, MO, USA). All cell lines had their identity confirmed by short tandem repeat (STR) profiling by the PowerPlex® 16 HS System kit (Promega, Madison, WI, USA), and were routinely tested for mycoplasma contamination by PCR amplification.

For functional assays, cells were seeded in six-well uncoated plates at a density of 5x10^5^ cells/well in complete growth medium and allowed to attach for 24h. In all performed assays, cells were treated with 10 µg/mL trastuzumab (Herceptin®, Roche Basel, Switzerland, 21 mg/mL stock concentration) in FBS-deprived growth medium for 24h or 120h, unless otherwise specified, and/or stimulated with 10 ng/mL of human recombinant EGF (Merck Millipore, Burlington, MA, USA) for 10 min immediately prior to cell lysis. All experiments were performed in triplicate. As a negative control of trastuzumab treatment (untreated condition), 10 µg/mL of a mouse IgG1 isotype control (R&D Systems, Minneapolis, MN, USA) was used.

**CRISPR/Cas9 genomic silencing of *ST6GAL1***

The CRISPR/Cas9 knock-out (K.O.) of the *ST6GAL1* gene in the NCI-N87 GC cell line was performed as previously described [17]. A validated guide RNA (gRNA), with the sequence TGTATCCTCAAGCAGCACCC, targeting the exon 1 of the *ST6GAL1* locus was used [18]. NCI-N87 cells were co-transfected with a plasmid containing the gRNA sequence (deposit 106714, Addgene) and a Cas9-GFP plasmid (deposit 68371, Addgene). Following cell transfection, fluorescence-activated cell sorting (FACS) was performed to isolate single-cell isogenic K.O. clones. Indels within the target genomic locus were firstly analyzed by IDAA PCR, followed by fragmentation analysis. Three clones were validated and selected for the following experiments. Validation of the detected indels in the selected clones was performed by direct Sanger sequencing and using the tracking of indels by decomposition (TIDE) bioinformatic tool [19].

**Real-Time Quantitative PCR**

Total RNA was extracted from subconfluent cell cultures using the TRI Reagent (Sigma-Aldrich). 1 µg of total RNA was converted into cDNA using the SuperScript® Reverse Transcriptase (Invitrogen^TM^, Carlsbad, CA, USA), according to manufacturer’s instructions. *ST6GAL1* mRNA expression was quantified by real-time quantitative PCR, using the TaqMan^TM^ Universal PCR Master Mix II, no UNG and the Hs00949382 *ST6GAL1* TaqMan^TM^ gene expression assay (Applied Biosystems®, Foster City, CA, USA). The mRNA expression levels of *RNA18S5* (Hs99999901) endogenous control were also measured for normalization of target gene abundance. The acquired data were analyzed by the ΔΔ*C*_t_ method.

**Immunofluorescence**

Cells were grown onto 13 mm glass slides and fixed with 4% paraformaldehyde for 5 min at RT. Slides were permeabilized with 0.5% Triton^TM^ X-100 (Sigma-Aldrich) for 10 min at 4ºC. Cells were blocked with 1:5 dilution of either normal rabbit serum (Dako) in 10% BSA (ErbB2, ST6Gal1 and STn), or just 10% BSA (SNA) for 30 min at RT, and incubated with primary antibodies and lectins (Table S3) overnight (ON) at 4ºC. Cells were labeled with 1:500 Alexa Fluor^TM^ 488-conjugated secondary antibodies (Invitrogen^TM^) and 1:1000 streptavidin-FITC in a for 45 min at RT and underwent nuclear staining with DAPI (Sigma-Aldrich). Images were acquired with a Zeiss Axio cam MRm and the AxioVision Rel. 4.8 software (Zeiss).

**Western and lectin blotting**

Both subconfluent and treated cell cultures were washed twice with ice-cold phosphate-buffered saline (PBS) and directly collected in lysis buffer 17 (R&D Systems) supplemented with 1 mM sodium orthovanadate (Sigma-Aldrich), 1 mM phenylmethanesulfonylfluoride (Sigma-Aldrich) and protease inhibitor cocktail (Roche). Regarding ErbB2-positive gastric tumor specimens, frozen tissues were vigorously rinsed with ice-cold PBS and protein extraction was performed using RIPA buffer (750 µL per 100 mg of tissue) supplemented with dithiothreitol (DTT) and protease inhibitor cocktail, using a tissue homogenizer (Kimble Chase, Vineland, NJ, USA). Tissues were further sonicated for 5 min at medium intensity and centrifuged at 12 000g for 15 min at 4ºC. Supernatants were collected. Total protein concentrations of whole cell lysates were determined using the DC protein assay kit (BioRad, Hercules, CA, USA). Equal amounts of protein were separated by SDS-PAGE and blotted onto nitrocellulose membranes (GE Healthcare, Chicago, IL, USA). Following 1h blocking at RT, membranes were incubated with primary antibodies and lectins (Table S3) ON at 4ºC. Then, membranes were incubated with horseradish peroxidase-labeled secondary antibodies (Jackson Immunoresearch, Cambridgeshire, United Kingdom) or streptavidin (GE Healthcare) for 1h at RT. Chemiluminescence signals were obtained using the ECL WB detection reagent and films (GE Healthcare). Densitometry was performed using the Quantity One® 1-D analysis software (BioRad). Relative signal quantification was performed by scanning densitometry of the α-tubulin/β-actin-normalized target protein band.

**Immunoprecipitation and Peptide *N*-Glycosidase F digestion**

A total protein amount of 400 µg from NCI-N87 whole cell lysates, or 1000 µg from fresh frozen ErbB2-positive gastric tumor lysates, were pre-cleared with 30 µL of Protein G Sepharose 4 Fast Flow beads (GE Healthcare) for 2h at 4ºC. In parallel, 5 µL of anti-ErbB2 mAb clone 29D8 (Cell Signaling Technology, Danvers, MA) was coupled to 60 µL of beads. ErbB2 immunoprecipitation was performed by incubating the pre-cleared lysates with the antibody-conjugated beads ON at 4ºC with gentle rotation. Normal rabbit IgGs (Merck Millipore) were used as a negative immunoprecipitation control. Non-specific binding was eliminated by washing the beads with 0.1% Triton^TM^ X-100. The immune complexes were released and denaturated by boiling for 10 min in Laemmli buffer (BioRad) containing 5% β-mercaptoethanol at 100°C. Then, SDS-PAGE separation and Western blot were performed as described.

Total *N*-glycan release from bead-conjugated immune complexes was accomplished through Peptide *N*-Glycosidase F (PNGase F) (New England BioLabs® Inc., Ipswich, MA, USA) digestion according to manufacturer’s instructions. Briefly, immunoprecipitates were incubated with denaturating buffer for 10 min at 100°C, and digested with 2 units of PNGase F ON at 37°C. The digestion reaction was stopped by boiling the beads for 10 min in Laemmli buffer containing 5% β-mercaptoethanol at 100°C.

**Flow cytometry**

The cell surface expression of various terminal glycan antigens was determined through the flow cytometric staining of NCI-N87 GC cells with distinct lectins and monoclonal antibodies (mAbs). Briefly, subconfluent (10^5^ cells/antigen) cell cultures were detached and stained with lectins and mAbs (Table S3) for 30 min on ice. Cells were then labeled with FITC-conjugated streptavidin, for lectin detection, or secondary anti-mouse Alexa Fluor^TM^ 488-conjugated antibody (Invitrogen^TM^), for mAb detection, for 30 min on ice. Cells were strained, labeled with propidium iodide (PI) and measured using a BD FACSCanto^TM^ II instrument (BD Biosciences, San Jose, CA, USA). Data were analyzed using FlowJo software (BD Biosciences).

The cell surface expression of ErbB2 was determined through the flow cytometric staining of NCI-N87 GC cells with trastuzumab. Briefly, cell cultures treated with 10 µg/mL of trastuzumab for 12h and 24h were detached and stained with freshly prepared 10 µg/mL trastuzumab for 30 min on ice. Cells were then labelled with secondary anti-human Alexa Fluor^TM^ 488-conjugated antibody (Invitrogen^TM^), for ErbB2 detection, for 30 min on ice.

Cell proliferation was determined by flow cytometric measurement of bromodeoxyuridine (BrdU) incorporation. Briefly, cell cultures in exponential growth phase were incubated with 10 µM BrdU diluted in complete growth medium for 1h at 37ºC in an atmosphere of 5% CO_2_. Cell cultures were then detached and fixed in ice-cold methanol for 30 min on ice. Fixed cells were incubated with 4M hydrochloric acid for 20 min at RT, to achieve denaturation of double-stranded DNA. Cells were incubated with anti-BrdU primary antibody (clone Bu20a, Dako), diluted 1:20 in PBS 0,5% Tween® 20 0,05% BSA for 1h at RT, and with FITC-conjugated anti-mouse secondary antibody (Dako) for 30 min at RT.

To assess trastuzumab-induced cell death, treated cell cultures were detached and stained with the Annexin V-FITC Apoptosis detection kit (Invitrogen^TM^), according to manufacturer’s instructions. The appropriate single staining (for the purpose of fluorescence compensation) and secondary antibody controls were included in all experiments.

**Capillary electrophoresis-electrospray ionization-mass spectrometry glycomic analysis of ErbB2**

ErbB2 immunoprecipitates from NCI-N87 whole cell lysates and fresh frozen ErbB2-positive gastric tumors were separated by SDS-PAGE on NuPAGE^TM^ 4-12% Bis-Tris Protein Gels (Invitrogen^TM^) for sample clean-up. Protein gels were stained for 3h with the Colloidal Blue staining kit (Invitrogen^TM^) and rinsed with distilled water ON. A strong ~185 kDa band was excised for further protein identification and glycomic and glycoproteomic analysis. Capillary electrophoresis-electrospray ionization-mass spectrometry (CE-ESI-MS) analysis of ErbB2 total released *N*-glycans was performed as described previously [37]. Briefly, total *N*-glycan release was accomplished by in-gel digestion with 2 units of PNGase F (Roche) in 2% NP-40, ON at 37ºC, and was followed by sialic acid linkage-specific derivatization, resulting in the ethyl esterification of α2,6NeuAc (derivatized mass: 319.127 Da) and the amidation of α2,3NeuAc (derivatized mass: 290.111 Da). Total released *N*-glycans were incubated with 20 µL ethyl esterification reagent (250 mM EDC and 250 mM HOBt in ethanol) for 30 min at 37ºC. The reaction mixture was further incubated with 4 µL 28% NH_4_OH for 30 min at 37ºC for the stabilization of α2,3-linked sialic acids. Derivatized *N*-glycans were purified by cotton hydrophilic interaction liquid chromatography solid-phase extraction (HILIC SPE). Samples were eluted in 10 µL of miliQ water and dried by vacuum concentration. Then, total released, derivatized and purified *N*-glycans were labelled at the reducing end with the Girard’s reagent P (GirP) (50 mM GirP in 90% ethanol and 10% glacial acetic acid) for 1h at 60ºC. Following GirP labeling, samples were dried by vaccum concentration at 60ºC and resuspended in 11 µL leading electrolyte of 200 mM ammonium acetate pH = 4.0 for sheathless CE-ESI-MS analysis in a CESI 8000 system (Sciex, Farmingham, MA, USA) coupled to an Impact qTOF mass spectrometer using a neutral coated capillary. All experiments were performed in positive-ionization mode using the following parameters: capillary voltage 1200 V, drying gas temperature 150 ºC, drying gas flow rate 1.2 L/min. Profile spectra were acquired using mass range *m/z* = 150 to *m/z* = 2000. Fragmentation was performed at 1.00 Hz on the three most abundant precursor ions in a range of m/z 550–2000. The collision energies were set as a linear curve in a m/z dependent manner, ranging from 55 eV at m/z 700 to 124 eV at m/z 1800 for all charge states (1–5), applying a basic stepping mode. Glycan species molecular masses were determined, and relative quantification was performed over the averaged deconvoluted mass spectra of the full peak using the relative signal intensities of each species. Data were analyzed with the Compass Data Analysis software version 4.2. (Bruker, Billerica, MA, USA).

**Liquid chromatography tandem mass spectrometry glycoproteomic analysis of ErbB2**

Isolation of ErbB2-containing gel bands was performed as described above. Briefly, excised bands were reduced with 10 mM DTT, alkylated with 50 mM iodoacetamide and in-gel digested with trypsin using a Proteineer DP digestion robot (Bruker). Tryptic peptides were analyzed by liquid chromatography tandem mass spectrometry (LC-MS/MS), via on-line C18-nano-HPLC-MS with a system consisting of an Easy nLC 1000 gradient HPLC system (Thermo Fisher Scientific), and a Orbitrap Fusion^TM^ Lumos^TM^ Tribrid^TM^ (Thermo Fisher Scientific) mass spectrometer. Fractions were injected onto a homemade pre-column (100 μm × 15 mm; Reprosil-Pur C18-AQ 3 μm, Dr. Maisch, Ammerbuch, Germany) and eluted via a homemade analytical nano-HPLC column (15 cm × 50 μm; Reprosil-Pur C18-AQ 3 um). The gradient was run from 0% to 50% solvent B (100/0.1 water/formic acid FA v/v) in 20 min. The nano-HPLC column was drawn to a tip of ∼5 μm and acted as the electrospray needle of the MS source. MS/MS spectra were acquired in data-dependent mode (top-10) with a normalized collision energy of 32% and recording of the MS/MS spectrum in the Orbitrap. To increase the fragmentation of *N*-glycosylated peptides, the HexNAc oxonium ion at *m/z* = 204.087 was set as Product Ion Trigger, and three additional MS/MS scans of the corresponding precursor ion were performed with higher-energy collisional dissociation (HCD) normalized collision energies of 32, 37 and 41%, respectively, and a collision-induced dissociation (CID) energy of 35 V.

In a post-analysis process, raw data were first converted to peak lists using Proteome Discoverer version 2.2 (Thermo Electron), and then submitted to the Uniprot Homo sapiens database (67911 entries), using Mascot v. 2.2.04 (www.matrixscience.com) for protein identification. Mascot searches were with a tolerance of 5 and 20 ppm for precursor and fragment ions, respectively, and trypsin was selected as the proteolytic enzyme. Up to two missed cleavages were allowed. Methionine oxidation and protein *N*-terminal acetylation were set as a variable modification and carbamidomethyl on cysteines was set as a fixed modification. The data was also analyzed using Byonic^TM^ version 2.13.2 (Protein Metrics, Cupertino, CA, USA) using default settings [38]. In addition to the settings for common modifications as described above, the “*N*-glycan 309 mammalian no sodium” database was used for the search and assignment of *N*-glycosylated peptides. Glycopeptide assignment was performed by manual spectra interpretation using the Xcalibur^TM^ software (Thermo Fisher Scientific).

**Metabolic activity assay**

Trastuzumab-induced cytotoxicity in NCI-N87 GC cells was evaluated with a resazurin metabolic activity assay. Briefly, in metabolically active mitochondria, the resazurin dye is reduced to the fluorescent resorufin metabolite (excitation – 530 nm, emission – 590 nm). NCI-N87 GC cells were seeded, in triplicate, at a density of 4x10^3^ cells/well in 96-well uncoated plates and allowed to attach for 24h in complete growth medium. Cells were then treated with increasing concentrations of trastuzumab (0.01 – 100 µg/mL) in FBS-deprived growth medium for 120h. Cells treated with 1 mM H_2_O_2_ and 10 µg/mL of a mouse IgG1 isotype control were included as positive and negative controls, respectively. Once completed the treatment period, cells were incubated with 0.01 mg/mL of resazurin (Sigma-Aldrich) for 3h at 37ºC. Quantification of resorufin-emitted fluorescence was performed in a Synergy^TM^ Mx microplate reader (Agilent, Santa Clara, CA, USA).

**ErbB2 protein half-life assay**

A cycloheximide (CHX) blocking assay was performed to determine ErbB2 protein half-life. Cells were seeded and allowed to attach for 24h in complete growth medium, and subsequently treated with 80 µg/mL CHX (Sigma-Aldrich), a protein synthesis inhibitor, for 0h, 12h, 24h, 48h, 72h and 96h. Whole cell lysates were collected and Western blot analysis of ErbB2 was performed as described. ErbB2 protein half-life was calculated based on the density of the β-actin-normalized ErbB2 Western blot bands, using the one phase exponential decay function, and was defined as the time required for the ErbB2 protein to decrease to 50% of its initial level.

**Cell surface protein biotinylation assay**

The levels of membrane-bound and cytosolic ErbB2 were determined using the EZ-Link™ Sulfo NHS-SS Biotinylation Kit (Thermo Fisher Scientific), according to manufacturer’s instructions. Briefly, cell surface proteins from trastuzumab-treated cell cultures were labelled with 1mM of Sulfo-NHS-SS-Biotin in ice-cold PBS (supplemented with 0,1mM CaCl_2_ and 1mM MgCl_2_) for 30 min on ice. Non-bound biotin was quenched with 0.1M glycine for 10 min on ice. Whole cell lysates were prepared and quantified as previously described. For the pull-down of biotinylated cell surface proteins, 100 µg of whole cell lysates were incubated with streptavidin-agarose beads ON at 4ºC with gentle rotation. Samples were centrifuged, and non-biotinylated proteins were recovered in the supernatant. Non-specific binding was eliminated by washing the beads with 1% Triton^TM^ X-100. Biotinylated complexes were released and denaturated by boiling for 10 min in Laemmli buffer (BioRad) containing 5% β-mercaptoethanol at 100°C. Then, SDS-PAGE separation and Western blot analysis of ErbB2 and the mitochondrial marker cytochrome c were performed as described.

**Cross-linking assay**

Cells were seeded and allowed to attach for 24h in complete growth medium, and subsequently treated with 10 µg/mL of trastuzumab in FBS-deprived growth medium for 24h. Treated cell cultures were washed twice with ice-cold PBS and incubated with 2.5 mM of the cross-linking reagent bis(sulfosuccinimidyl)suberate (BS3) (Sigma-Aldrich) in PBS for 3h on ice. The cross-linking reaction was quenched with 10 mM of Tris-HCl for 15 min on ice. Whole cell lysates were collected and Western blot analysis of ErbB2, EGFR, ErbB3 and ErbB4 was performed as described.

**Kinase phospho-arrays**

The relative phosphorylation levels of 43 kinase and 49 receptor tyrosine kinase (RTK) phosphorylation sites (Table S4) were determined with the Human Phospho-Kinase/RTK Array kits (ARY003B and ARY001B, respectively, R&D Systems), according to manufacturer’s instructions. Briefly, treated cell cultures of NCI-N87 WT and a representative *ST6GAL1* K.O. clone (C1) were lysed as described, and 400 µg and 200 µg of protein from whole cell lysates were used, respectively. Chemiluminescence signals were obtained using the ECL Western blot detection reagent and films (GE Healthcare). Densitometry was performed using the Quantity One® 1-D analysis software (BioRad).

**Bioinformatic analysis**

The log_2_ median-centered gene expression data of human GC patients were extracted from the Oncomine^TM^ platform (www.oncomine.org) [39]. The association analysis was performed in GraphPad Prism 7 using the DErrico dataset [40]. The DErrico dataset comprises expression data of 36 malignant primary gastric tumors obtained by Affymetrix GeneChip Human Genome U133A/B. The *ERBB2* (Affymetrix probe: 210930_s_at) and *ST6GAL1* (Affymetrix probe: 214971_s_at) data were not normally distributed (determined by Shapiro–Wilk normality test) and association was therefore analyzed using Spearman’s correlation analysis.

**Statistical analysis**

Quantitative data is presented as mean ± SD of three independent replicates. All statistical differences between NCI-N87 WT and three isogenic *ST6GAL1* K.O. clones were determined using one-way ANOVA analysis of variance. Data populations were verified to follow a Gaussian distribution and to have similar variance. ErbB2 protein half-life was calculated using the one phase exponential decay function. All statistical tests were performed using the GraphPad Prism 7 software. *P* < 0.05 was considered statistically significant (**P* < 0.05, ***P* < 0.01, and ****P* < 0.001).
