# Supplementary material for: ST6Gal1 targets the ectodomain of ErbB2 in a site-specific manner and regulates gastric cancer cell sensitivity to trastuzumab
Source: Oncogene. 2021 May 4;40(21):3719–33. doi: 10.1038/s41388-021-01801-w (PMC8154592; doi:10.1038/s41388-021-01801-w)
Supplement: Supplementary file 11 — Supplementary Figures and Table Legends [file 41388_2021_1801_MOESM11_ESM.docx]

**Supplementary Information**

**Figure legends and tables**

**Figure S1. Validation of CRISPR/Cas9-induced indels at the *ST6GAL1* locus in three isogenic clones of ErbB2-positive NCI-N87 gastric cancer cells.** **A** Indel detection by amplicon analysis (IDAA) PCR on the amplicon harboring the locus targeted by the *ST6GAL1* guide RNA (gRNA) in NCI-N87 gastric cancer (GC) WT cells and three isogenic *ST6GAL1* K.O. clones; **B** Indel signature confirmation by direct Sanger sequencing of the *ST6GAL1* amplicon; **C** *ST6GAL1* indel quantification and +1 overhang nucleotide base characterization using the tracking of indels by decomposition (TIDE) web tool; **D** Analysis of *ST6GAL1* mRNA expression in NCI-N87 WT and *ST6GAL1* K.O. GC cells; **E** Cell surface expression of α2,6NeuAc in NCI-N87 WT and *ST6GAL1* K.O. GC cells, analyzed by flow cytometry with the *Sambucus nigra* agglutinin (SNA); **F** Assessment of WT and *ST6GAL1* K.O. cell proliferation by flow cytometric measurement of bromodeoxyuridine (BrdU) incorporation; n = 3 (mean ± SD); Comparisons were made using one-way ANOVA analysis of variance (n = 3; mean ± SD; **p* < 0.05; ***p* < 0.01; ****p* < 0.001); n.s. – non-significant; C1 – clone 1; C2 – clone 2; C3 – clone 3.

**Figure S2. Workflow for the structural characterization and site-specific mapping of ErbB2 *N*-glycan chains.** ErbB2 was immunoprecipitated from NCI-N87 WT and *ST6GAL1* K.O. cells whole cell lysates, separated by SDS-PAGE and stained with Colloidal Blue. A prominent ~185 kDa band, corresponding to the fully glycosylated ErbB2, was selected and excised for further mass spectrometry (MS)-based protein identification, and glycomic (right arm) and glycoproteomic (left arm) analysis; LC-MS/MS – liquid chromatography tandem mass spectrometry; HILIC - hydrophilic interaction liquid chromatography; GirP – Girard’s reagent P; CE-MS – capillary electrophoresis mass spectrometry.

**Figure S3. *ST6GAL1* K.O. induces the enrichment of ErbB2 N530 glycosite in terminally fucosylated species.** **A** Upper panels – higher energy collision dissociation (HCD) MS/MS spectra of the N530 glycopeptide modified with a di-sialylated (dS) biantennary *N*-glycan in WT ErbB2, and with a di-fucosylated (dF) biantennary *N*-glycan in *ST6GAL1* K.O. C3 ErbB2; Lower panel – collision-induced dissociation (CID) MS/MS spectrum of the N530 glycopeptide modified with a dF biantennary *N*-glycan in *ST6GAL1* K.O. C3 ErbB2; **B** Left panel – Combined extracted ion chromatogram of the N530 glycopeptide modified with: a tri-fucosylated (tF) biantennary *N*-glycan (peak 1, peptide+GlcNAc(4)Man(3)Gal(2)Fuc(3), ion at *m/z* 1441.250, [M+3H]^3+^), a mono-sialylated mono-fucosylated (mSmF) biantennary *N*-glycan (peak 2, peptide+GlcNAc(4)Man(3)Gal(2)Fuc(1)NeuAc(1), ion at *m/z* 1440.910, [M+3H]^3+^) and dS biantennary *N*-glycan biantennary *N*-glycan (peak 3, peptide+GlcNAc(4)Man(3)Gal(2)NeuAc(2) ion at *m/z* 1489.256, [M+3H]^3+^) in WT and *ST6GAL1* K.O. C3 ErbB2; C1 – clone 1; C2 – clone 2; C3 – clone 3.

**Figure S4. ErbB2 N124 glycosylation site is modified with oligomannosidic *N*-glycan chains in gastric cancer clinical specimens.** **A** Immunohistochemical detection of ErbB2 in an intestinal-type gastric carcinoma; **B** ErbB2 immunoprecipitation from the corresponding fresh frozen tissue specimen of the ErbB2-positive tumor depicted in (A); **C** Higher energy collision dissociation (HCD) MS/MS fragmentation spectra and isotopic distribution of the N124 glycopeptide modified with the Man8 oligomannosidic *N*-glycan in an ErbB2-positive intestinal-subtype gastric carcinoma; **D** Schematic representation of glycosylation site assignment and structural characterization in the same ErbB2-positive gastric carcinoma sample.

**Figure S5. Kinase phosphorylation status of untreated NCI-N87 cells.** **A** Target protein coordinate template for the Human Phospho-Kinase Array kit membrane; **B** Phosphorylation status of 43 human kinases in untreated NCI-N87 WT and *ST6GAL1* K.O. cells; **C** Relative (fold change) quantification of the phosphorylation levels of 43 human kinases in untreated *ST6GAL1* K.O. C1 sample, in comparison with the WT control (reference); C1 – clone 1.

**Table S1.** Clinicopathological data and glycan biomarker expression in ErbB2-positive gastric cancer clinical samples.

**Table S2.** Glycoproteomic data from ErbB2.

**Table S3.** Specification of primary antibodies and lectins.

**Table S4.** Human Phospho-Kinase/RTK Array Membrane Coordinates.
